# Metabolic and enzymatic elucidation of cooperative degradation of red seaweed agarose by two human gut bacteria

**DOI:** 10.1038/s41598-021-92872-y

**Published:** 2021-07-06

**Authors:** Eun Ju Yun, Sora Yu, Na Jung Park, Yoonho Cho, Na Ree Han, Yong-Su Jin, Kyoung Heon Kim

**Affiliations:** 1 Department of Biotechnology, Graduate School, Korea University, Seoul, 02841 Republic of Korea; 2 Carl R. Woese Institute for Genomic Biology, University of Illinois at Urbana-Champaign, Urbana, IL 61801 USA; 3 Department of Food Science and Human Nutrition, University of Illinois at Urbana-Champaign, Urbana, IL 61801 USA

**Keywords:** Applied microbiology, Environmental microbiology

## Abstract

Various health beneficial outcomes associated with red seaweeds, especially their polysaccharides, have been claimed, but the molecular pathway of how red seaweed polysaccharides are degraded and utilized by cooperative actions of human gut bacteria has not been elucidated. Here, we investigated the enzymatic and metabolic cooperation between two human gut symbionts, *Bacteroides plebeius* and *Bifidobacterium longum* ssp*. infantis*, with regard to the degradation of agarose, the main carbohydrate of red seaweed. More specifically, *B. plebeius* initially decomposed agarose into agarotriose by the actions of the enzymes belonging to glycoside hydrolase (GH) families 16 and 117 (i.e., *Bp*GH16A and *Bp*GH117) located in the polysaccharide utilization locus, a specific gene cluster for red seaweed carbohydrates. Then, *B. infantis* extracted energy from agarotriose by the actions of two agarolytic β-galactosidases (i.e., Bga42A and Bga2A) and produced neoagarobiose. *B. plebeius* ultimately acted on neoagarobiose by *Bp*GH117, resulting in the production of 3,6-anhydro-l-galactose, a monomeric sugar possessing anti-inflammatory activity*.* Our discovery of the cooperative actions of the two human gut symbionts on agarose degradation and the identification of the related enzyme genes and metabolic intermediates generated during the metabolic processes provide a molecular basis for agarose degradation by gut bacteria.

## Introduction

One of the crucial roles of the gut microbiota is to extract energy and nutrients from dietary polysaccharides using Carbohydrate-Active enZymes (CAZymes) that are not expressed by the human body^1–3^. Various oligosaccharides generated from the degradation of dietary polysaccharides by gut bacteria markedly impact the health and gut physiology of the host by modulating the composition of the gut microbiota^4^. Two phyla—Bacteroidetes and Firmicutes—dominate the bacterial community of the adult distal gut^5^. In particular, members of the genus *Bacteroides* are broadly involved in degrading host glycans^6,7^ and terrestrial plant polysaccharides in the gut^1,2,4,8^. For example, *Bacteroides thetaiotaomicron*—a prominent human gut symbiont—contains a huge repertoire of glycoside hydrolases (GHs) and polysaccharide lyases for degrading dietary polysaccharides derived from plants, such as starch, pectin, and hemicellulose^1,4,9^. Most studies regarding the degradation pathways and roles of dietary polysaccharides in human and animal guts have focused on polysaccharides derived from terrestrial plants^2,8,10^. However, the degradation pathways of dietary seaweeds in the gut and the potential roles of oligosaccharides and rare sugars generated after the consumption of seaweeds have rarely been studied to date^3,11,12^.

In East Asia including Japan, Korea, and China, people frequently consume red or brown seaweeds, such as agar, nori, kombu, and wakame^13^. These seaweeds have potential therapeutic or preventative effects against various diseases and health concerns such as Type 2 diabetes^14^, obesity^15^, cardiovascular disease mortality^16^, and hypocholesterolaemic effect^17^. Because the major components of seaweeds are polysaccharides^18,19^, it is likely that the health benefits of seaweed diet may be attributed to the polysaccharides. For example, fucoidan, one of the major polysaccharides of brown seaweeds, is widely known to be the key for the anticancer activity of brown seaweeds^20–23^. With regard to the health benefits of red seaweeds, 3,6-anhydro-l-galactose (AHG), the monomeric sugar of agar along with galactose, was reported to exhibit strong in vitro﻿ anti-inflammatory activity, indicating the significant suppression of nitrite production in our previous study^24^. Therefore, we reasoned that AHG may have been responsible for the beneficial effects of dietary red seaweeds reported in previous clinical trials and epidemiological studies^25–27^. However, it is debatable if and how AHG is released in the human gastrointestinal tract after red seaweeds are ingested. Agar is the major component of red seaweeds, and is mainly composed of agarose, which is the linear polysaccharide of AHG and d-galactose joined alternately by α-1,3- and β-1,4-glycosidic linkages^28^. Because agar cannot be degraded by human GHs, the degradation and metabolic pathways of agar in the human gut may rely on microbial GHs.

**Figure 1:**
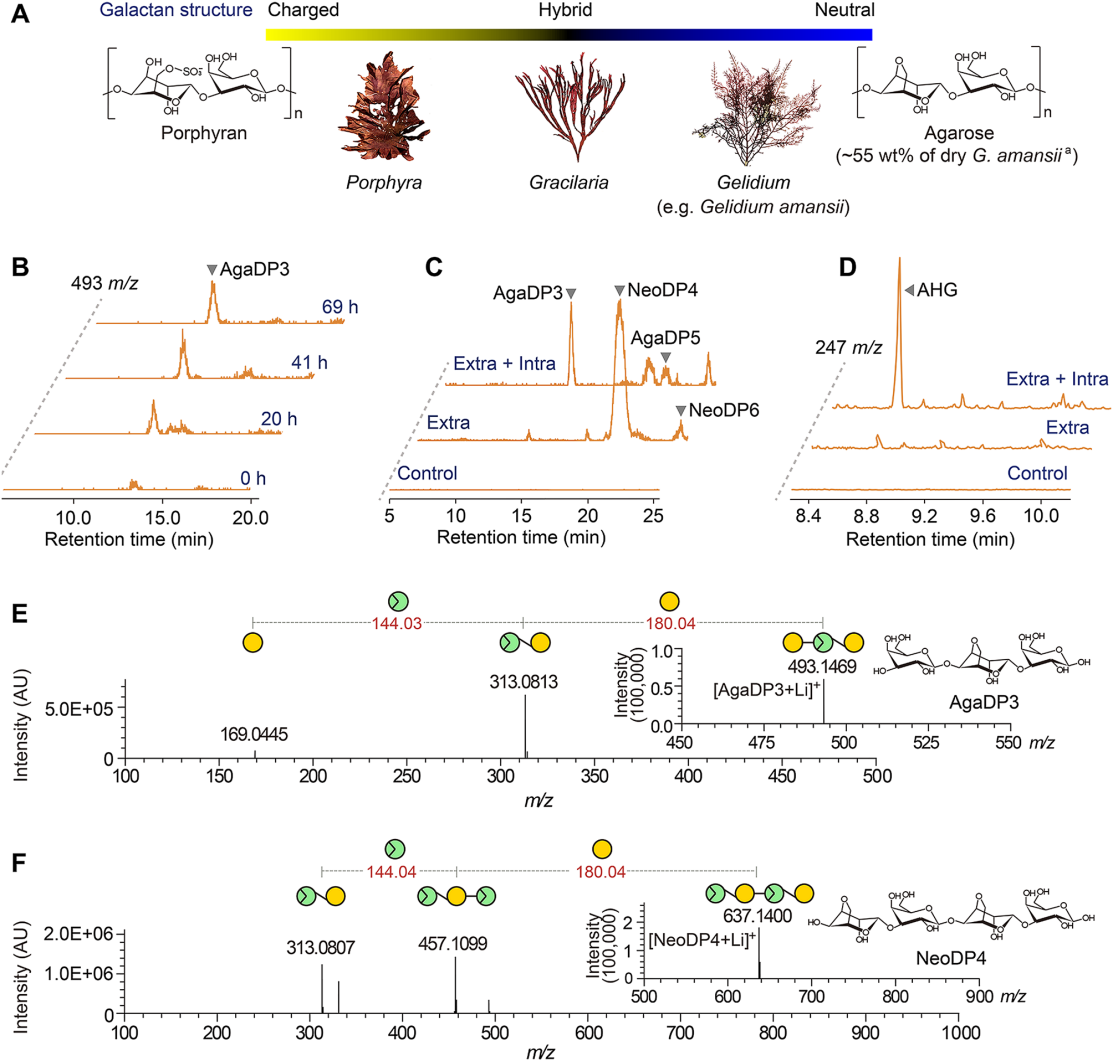
Agarose degradation profiles of *B. plebeius*. (**A**) Galactan structures found in various species of agarophytic red seaweeds (e.g., those from the genera *Porphyra*, *Gracilaria*, and *Gelidium*). Porphyran and agarose are the major carbohydrate components of red seaweeds. l-Galactose-6-sulfate and d-galactose are the monomeric constituents of porphyran, and AHG and d-galactose are the monomeric constituents of agarose. The main cell wall carbohydrates of species of the genera *Porphyra* and *Gelidium* are porphyran and agarose, respectively. Specifically, the main cell wall carbohydrate in red macroalgae (e.g., *Gelidium amansii*) is agar, which consists of agarose and agaropectin. (**B**) Profiles of agarose degradation by *B. plebeius.* During the fermentation of pretreated agarose by *B. plebeius*, AgaDP3—a trisaccharide composed of d-galactose and AHG—was produced as the main degradation product of agarose. We analyzed the AgaDP3 in the culture supernatant of *B. plebeius* by LC/MS − IT − TOF at 493 *m/﻿z*. (**C** and **D**) Analyses of the reaction products obtained from the sequential enzymatic reactions using extracellular crude enzymes (Extra in C and D) and cell free lysate containing intracellular and membrane-associated enzymes (Intra in C and D) of *B. plebeius* with agarose as the substrate. The AOSs and NAOSs were analyzed by LC/MS − IT − TOF (**C**), and the AHG produced by the enzymatic reactions was analyzed by GC–MS at 247 *m﻿/z﻿* (**D**). (E and F) Identification of AgaDP3 and NeoDP4 using LC/MS − IT − TOF. Tandem mass spectra of AgaDP3 (**E**) and NeoDP4 (**F**). The inset figures of E and F show the mass spectra of AgaDP3 and NeoDP4, respectively.

The CAZymes of only two human gut bacteria—namely *Bacteroides plebeius* DSM 17135 and *Bacteroides uniformis* NP1—have been reported to degrade agar^11,29^. Specifically, *B. plebeius* was isolated from the guts of seaweed-eating Japanese people and was found to possess CAZymes that act on red seaweed polysaccharides including porphyran (highly sulfated and methylated agarose)^30^ and agarose (non-sulfated agarose) (Fig. 1A)^3,29^. In particular, the CAZymes of *B. plebeius* are capable of degrading agarose^3,29,31^, the main polysaccharide of agar (Fig. 1A). However, *B. plebeius* is incapable of completely degrading and utilizing agarose as a carbon source, probably owing to the incompleteness of its agarose degradation system^29^. Therefore, agarose degradation in the human gut cannot be fully attributed to the agar-degrading enzymes of *B. plebeius* alone. As such, for dietary agarose to be further degraded into its monomeric sugars (AHG and galactose) in the human gut, other human gut bacteria must cooperate with *B. plebeius*. In this study, we have designed a model system consisting of agarose with *B. plebeius* together with a well-known human gut bacterium *Bifidobacterium longum* ssp*. infantis* ATCC 15697 that may be capable of cooperating in the degradation of agarose. Using this cooperative model system, the polysaccharide degradation pathway for red seaweed agarose by the possible human gut symbionts is provided.

## Results

### Agarose degradation profiles of *B. plebeius*

First, we cultured *B. plebeius* in a modified minimal broth^29^ supplemented with 5 g/L (w/v) agarose pretreated by a simulated gastric fluid for 2 h. We found that during the fermentation of *B. plebeius* with the pretreated agarose, agarotriose (AgaDP3; an agarooligosaccharide with a degree of polymerization (DP) of 3, composed of d-galactose, AHG, and d-galactose joined by β-1,4- and α-1,3-glycosidic linkages) was produced as the main fermentation product (Fig. 1B). The AgaDP3 generated by *B. plebeius* was identified by liquid chromatography/mass spectrometry hybrid ion trap time-of-flight (LC/MS − IT − TOF) analysis of the culture supernatant of *B. plebeius* grown on agarose (Fig. 1E).

Next, we investigated the agarose degradation pathway of *B. plebeius*—which leads to the accumulation of AgaDP3 from agarose—using in vitro﻿ reactions of crude enzymes obtained from *B. plebeius* grown with the pretreated agarose. The crude enzymes were divided into a fraction containing extracellular enzymes, and a cell-free lysate containing intracellular and membrane-associated enzymes. Initially, we mainly observed endo-type β-agarase activity in the fraction with extracellular enzymes (Supplementary Fig. 1A–C). The major reaction product with the extracellular enzymes was identified as neoagarotetraose (NeoDP4; an agarooligosaccharide with a DP of 4, composed of AHG, d-galactose, AHG, and d-galactose joined by alternating α-1,3- and β-1,4-glycosidic linkages) by LC/MS − IT − TOF analysis (Fig. 1F). The absence of neoagarobiose (NeoDP2; a disaccharide composed of AHG and d-galactose joined by an α-1,3-glycosidic linkage) in the reaction product of agarose with the extracellular enzymes indicated that exo-type β-agarase activity producing NeoDP2 did not occur in the extracellular enzyme fraction (Supplementary Fig. 1A–C). The intracellular enzyme fraction exhibited neoagarobiose hydrolase (NABH) activity, releasing AHG from NeoDP2 by cleavage of the α-1,3-glycosidic bond of NeoDP2 (Supplementary Fig. 2A–C). Finally, we confirmed AgaDP3 production resulting from the degradation of agarose by *B. plebeius* by the sequential reactions of the extracellular and intracellular enzymes of *B. plebeius*: agarose was initially depolymerized into NeoDP4 by the extracellular enzymes, and NeoDP4 was then hydrolyzed into AgaDP3 and AHG by the intracellular enzymes (Fig. 1C,D).

### Agarose-degrading enzymes in *B. plebeius*

The polysaccharide utilization locus (PUL) of *B. plebeius* contains CAZymes, which are active on algal polysaccharides including porphyran (highly sulfated and methylated agarose) and agarose (non-sulfated agarose)^29,31^. In the PUL of *B. plebeius*, the genes encoding enzymes belonging to GH families 16, 50, and 117 (i.e., *Bp*GH16A, *Bp*GH50, and *Bp*GH117) are related to agarose degradation pathway via the β-agarase system. Among these enzymes, *Bp*GH16A and *Bp*GH117 were biochemically characterized in the previous studies^29,31,32^. Here, we elucidated the combination and reaction sequence of enzymes leading to produce AgaDP3 and AHG from agarose, as we observed in the fermentation profiles of *B. plebeius* in the presence of agarose (Fig. 1B) and ﻿in vitro reactions using the crude enzymes of *B. plebeius* on agarose (Fig. 1C,D)*.*

**Figure 2:**
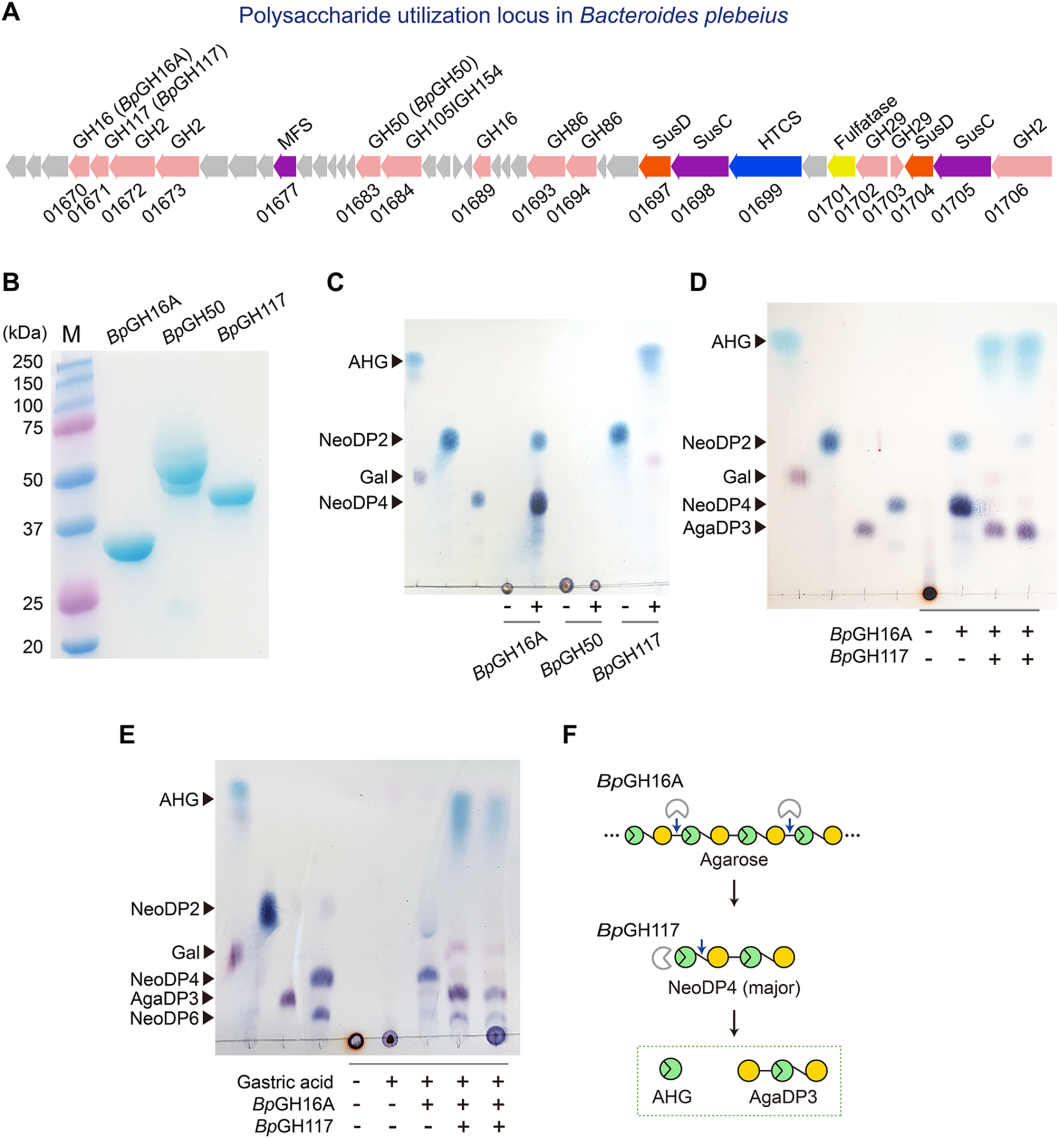
*Bp*GH16A and *Bp*GH117 are the key enzymes for the accumulation of AgaDP3 from agarose in *B. plebeius*. (**A**) The PUL of *B. plebeius*. Numbers below gene arrows are locus tag numbers without their prefix, BACPLE*.* Based on the sequence analysis, three genes located in the PUL of *B. plebeius*—i.e., BACPLE_01670, BACPLE_01671, and BACPLE_01683—encoding *Bp*GH16A, *Bp*GH117, and *Bp*GH50, respectively, are related to the agarolytic mechanism. Abbreviations: MFS, Major facilitator superfamily; HTCS, hybrid two-component system. (**B**) Purification of the agarose degradation pathway enzymes, *Bp*GH16A, *Bp*GH50, and *Bp*GH117, originating from *B. plebeius*. (**C**) Enzymatic activities of purified recombinant *Bp*GH16A, *Bp*GH50, and *Bp*GH117. The agarase activities of *Bp*GH16A and *Bp*GH50 were verified by the enzymatic reactions of each enzyme with agarose as the substrate. The NABH activity of *Bp*GH117 was verified by the enzymatic reaction with NeoDP2 as the substrate. The reaction products were analyzed by TLC. (**D**) Simulation of the agarolytic mechanism of *B. plebeius* using the sequential and the simultaneous enzymatic reactions of *Bp*GH16A and *Bp*GH117 with agarose*.* Lanes 1–4, the standards of the agar-derived sugars; lane 5, the agarose substrate; lane 6, the reaction products of *Bp*GH16A using agarose; lane 7, the reaction products of *Bp*GH117 with the reaction products of *Bp*GH16A; lane 8, simultaneous enzymatic reactions of *Bp*GH16A and *Bp*GH117 using agarose. (**E**) Effects of treatment of agarose with simulated gastric fluid on sequential or simultaneous hydrolysis of agarose using *Bp*GH16A and *Bp*GH117. The reaction products obtained from each step of agarose hydrolysis were analyzed by TLC. Lanes 1–3, the standards of agar-derived sugars, AHG, d-galactose (Gal), NeoDP2, AgaDP3, NeoDP4, and NeoDP6; lane 4, agarose substrate; lane 5, treatment of agarose using a simulated gastric fluid; lane 6, reaction products of *Bp*GH16A with agarose treated using a simulated gastric fluid; lane 7, reaction products of *Bp*GH117 with the reaction products of *Bp*GH16A; lane 8, reaction products obtained from the simultaneous reaction of *Bp*GH16A and *Bp*GH117 with agarose treated using a simulated gastric fluid. (**F**) Proposed mechanism of agarose degradation by *Bp*GH16A and *Bp*GH117 based on the results of in vitro enzymatic reactions.

After obtaining *Bp*GH16A, *Bp*GH50, and *Bp*GH117 via recombinant protein expression and purification of each (Fig. 2B), we performed the enzymatic reactions ﻿in vitro (Fig. 2C). First, we confirmed the activity of *Bp*GH16A on agarose substrate. As previously reported, the major reaction product of *Bp*GH16A with agarose was NeoDP4 (Fig. 2C)^32^, which was identified by combining the results of thin-layer chromatography (TLC) and LC/MS − IT − TOF analyses of the reaction products of *Bp*GH16A (Figs. 1F and 2C).

Second, we attempted to determine the activity of *Bp*GH50, which possibly takes part in the next step of the agarose degradation pathway by degrading neoagarooligosaccharides (NAOSs; agarooligosaccharides with AHG on the non-reducing end) into NeoDP2. As previously reported^33^, *Bp*GH50 did not exhibit an exo-type β-agarase activity producing NeoDP2 toward agarose (Fig. 2C). Moreover, we found that *Bp*GH50 did not exhibit activity on NeoDP4, the major product produced by *Bp*GH16A reaction with agarose (Supplementary Fig. 3). Finally, we confirmed the activity of *Bp*GH117, which is known to recognize AHG at the non-reducing end of NAOSs including NeoDP2 and produce AHG^31^, by TLC analysis of the reaction products of *Bp*GH117 (Fig. 2C). In summary, of the three enzymes related to the agarose degradation pathway, we found that only *Bp*GH16A and *Bp*GH117 participated in the agarose degradation (Fig. 2C).

The enzymatic reactions catalyzed both sequentially and simultaneously by *Bp*GH16A and *Bp*GH117 showed that agarose is degraded into AgaDP3 and AHG (Fig. 2D). These results are in good agreement with those obtained from the fermentation (Fig. 1B) and in vitro reactions using the crude enzymes of *B. plebeius* on agarose (Fig. 1C,D)*.* We also investigated the effect of a simulated gastric fluid on the agarose degradation pathway of *B. plebeius*. Although the treatment of agarose with a simulated gastric fluid might have partially cleaved the glycosidic linkages of agarose, as in the mild acid hydrolysis of agarose^34^, we obtained the same results with regard to the agarose degradation pathway and reaction products of *Bp*GH16A and *Bp*GH117 under the simulated gastric fluid conditions (Fig. 2E) as under in vitro﻿ enzymatic reaction conditions (Fig. 2D). Therefore, we suggest that in the agarose degradation pathway, *B. plebeius* accumulates AgaDP3 and AHG (Fig. 2F).

Meanwhile, although we did not verify which SusCD complex in *B. plebeius* is responsible for importing NeoDP4 into the periplasmic space, one of the two SusCD complexes, BACPLE 01697‒01698 and BACPLE 01704‒01705, found in the PUL of *B. plebeius* (Fig. 2A) might function as a transporter of NeoDP4 into the periplasmic space.

### AgaDP3 utilization by probiotic strain *B. infantis*

Because AgaDP3 was accumulated during the utilization of agarose by *B. plebeius*, as described above, we speculated on the metabolic fate of the accumulated AgaDP3 in the human gut. Agarooligosaccharides (AOSs; agarooligosaccharides with galactose on the non-reducing end) and NAOSs have prebiotic potential because they modulate the composition of the gut microbiota, thereby preventing gut dysbiosis^35^ and stimulating the growth of beneficial gut bacteria such as lactobacilli and bifidobacteria^36^.

**Figure 3:**
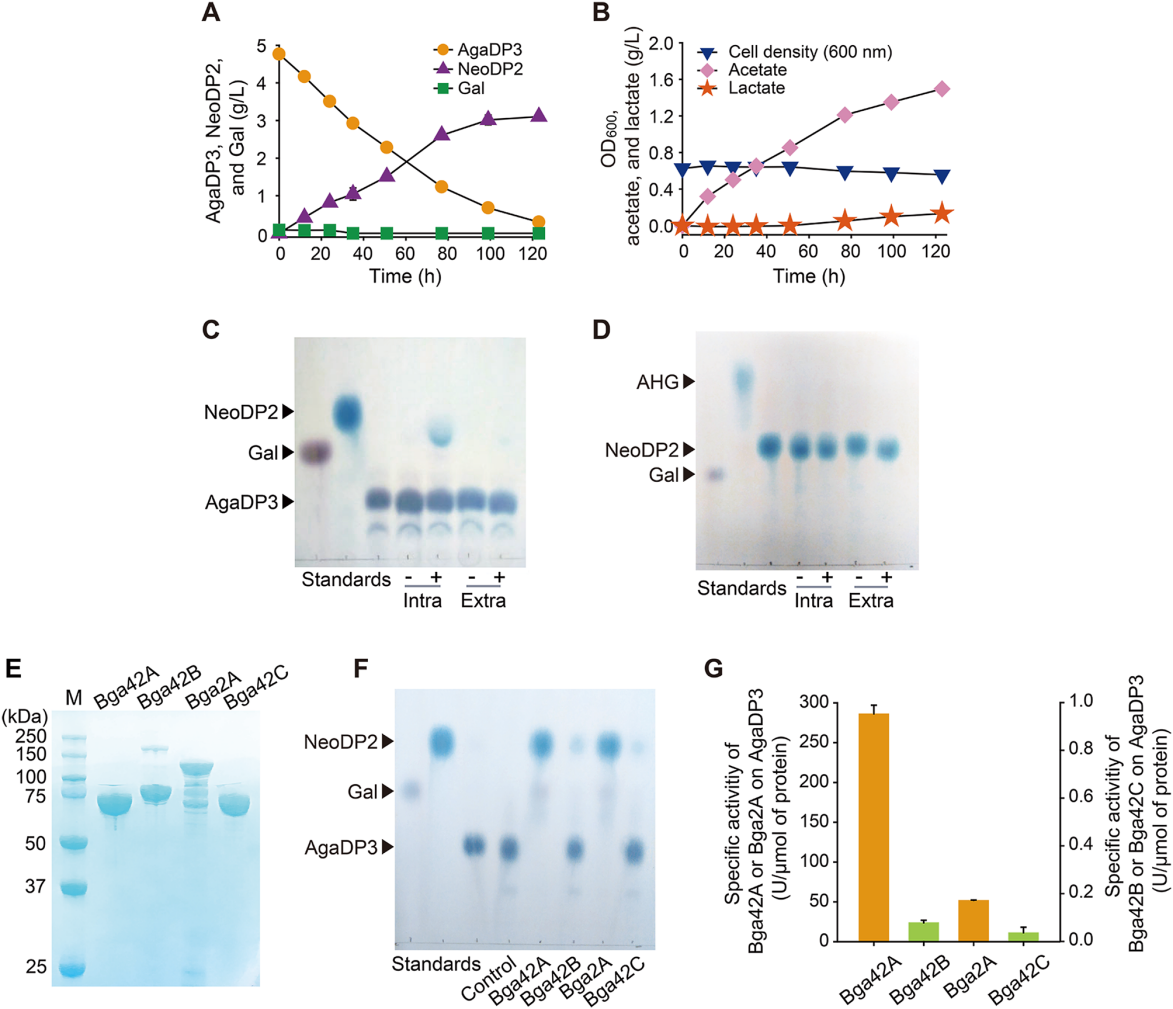
*B*. *infantis* extracts energy from AgaDP3 via its agarolytic β-galactosidase activity, and *B. plebeius* ultimately acts on NeoDP2, resulting in the production of AHG. (**A** and **B**) Time courses of AgaDP3 fermentation by *B. infantis.* Error bars mean ± S.D. (**C**) In vitro agarolytic β-galactosidase activities of extracellular crude enzymes (Extra) and cell-free lysate (Intra) obtained from *B. infantis* on AgaDP3. (**D**) In vitro NABH activities of extracellular crude enzymes (Extra) and cell-free lysate (Intra) obtained from *B. infantis* on AgaDP3. (**E**) SDS-PAGE analysis of the overexpressed and purified recombinant β-galactosidases Bga42A, Bga42B, Bga2A, and Bga42C originating from *B. infantis*. (**F**) TLC analysis of the reaction products of recombinant β-galactosidases from *B. infantis*—i.e., Bga42A, Bga42B, Bga2A, and Bga42C—with AgaDP3. (**G**) Comparison of the specific agarolytic β-galactosidase activities of the four β-galactosidases on AgaDP3. Error bars represent means ± S.D.

AgaDP3 was suggested as a prebiotic oligosaccharide in a previous study because of five distinct *Bifidobacterium* strains tested, *Bifidobacterium adolescentis* 1.2190 and *Bifidobacterium infantis* 1.2202 utilized AgaDP3^37^. However, the AgaDP3 utilization pathway adopted by these *Bifidobacterium* strains, and the key enzymes associated with AgaDP3 utilization have not been fully elucidated. As in a previous study^37^, AgaDP3 was also utilized by a well-known probiotic strain, *B. infantis* (Fig. 3A,B). Based on the observation of crude in vitro enzyme reactions, the AgaDP3 degradation pathway adopted by *B. infantis* is as follows. AgaDP3 is hydrolyzed into galactose and NeoDP2, probably by the action of the intracellular agarolytic β-galactosidases of *B. infantis* (Fig. 3C). Then, the galactose intracellularly produced from AgaDP3 is used by *B. infantis* as an energy source (Fig. 3A,B), whereas the produced NeoDP2 is not further degraded by *B. infantis* (Fig. 3D). The fermentation profiles revealed that most of AgaDP3 supplied as a carbon source was used for producing acetate than for the cell growth of *B. infantis* (Fig. 3B).

### AgaDP3-degrading β-galactosidases in *B. infantis*

The results obtained from the fermentation profiles of *B. infantis* under AgaDP3 condition (Fig. 3A) and in vitro crude enzyme reactions with AgaDP3 (Fig. 3C) demonstrated that *B. infantis* degrades AgaDP3 into NeoDP2 and d-galactose via its agarolytic β-galactosidase activity. *B. infantis* has five genes encoding β-galactosidase, however one of them (i.e., Blon_0268) was excluded because, according to the previous studies, Blon_0268 preferred β-1,6-linkages in β-1,6-d-galactobiose^38^. Therefore, we tested four genes encoding β-galactosidase, Blon_2016, Blon_2123, Blon_2334, and Blon_2416 corresponding to Bga42A, Bga42B, Bga2A, and Bga42C, respectively, to identify the enzymes responsible for the agarolytic β-galactosidase activity with regard to AgaDP3 in *B. infantis* (Fig. 3C)*.*

Four recombinant β-galactosidases originating from *B. infantis*—namely, Bga42A, Bga42B, Bga2A, and Bga42C—were prepared to test whether they could hydrolyze AgaDP3 (Fig. 3E). The functional expression of these β-galactosidases was verified by determining the β-galactosidase activities of the enzymes with regard to lactose (Supplementary Fig. 4). Interestingly, of the four recombinant β-galactosidases, Bga42A and Bga2A exhibited agarolytic β-galactosidase activity with regard to AgaDP3 (Fig. 3F,G). Although the two other enzymes, Bga42B and Bga42C, also showed agarolytic β-galactosidase activity, those specific activities were marginal as compared to those obtained from Bga42A and Bga2A (Fig. 3G).

To date, agarolytic β-galactosidase activity with regard to galactose residues at the non-reducing ends of AOSs including AgaDP3 has only been observed in a marine bacterium, *Vibrio* sp. EJY3, and a gut bacterium, *B. uniformis* NP1^11,39^. The β-galactosidases originating from *B. infantis* have been biochemically well-characterized using various substrates such as human milk oligosaccharides (HMOs) in the previous studies^38,40^; however, they have not been tested yet with respect to the degradation of agar-derived sugars such as AgaDP3. In the present study, we observed agarolytic β-galactosidase activities of Bga42A and Bga2A originating from *B. infantis, *a probiotic gut bacterium. Intriguingly, Bga42A is the first enzyme belonging to GH42 that exhibits an agarolytic β-galactosidase activity.

In the PUL of *B. plebeius*, the three genes, BACPLE_01672, BACPLE_01673, and BACPLE_01706, belong to GH2. Therefore, the agarolytic β-galactosidase activities of those purified recombinant proteins on AgaDP3 were measured in this study. The results revealed that among the three enzymes belonging to GH2, the recombinant protein of BACPLE_01672 showed the agarolytic β-galactosidase activity on AgaDP3 (Supplementary Fig. 5). However, the specific activity of the recombinant protein of BACPLE_01672 was significantly lower than that of Bga42A and Bga2A originating from *B. infantis* (Supplementary Fig. 5). In addition, during the fermentation of *B. plebeius* under the AgaDP3 condition, the concentration of AgaDP3 in the culture medium did not decreased (Supplementary Fig. 6A). The results of in vitro crude enzyme reactions showed that the crude enzymes of *B. plebeius* did not exhibit the agarolytic β-galactosidase activity on AgaDP3 (Supplementary Fig. 6B).

### Sequential co-culture of *B. plebeius* and *B. infantis* with pretreated agarose

**Figure 4:**
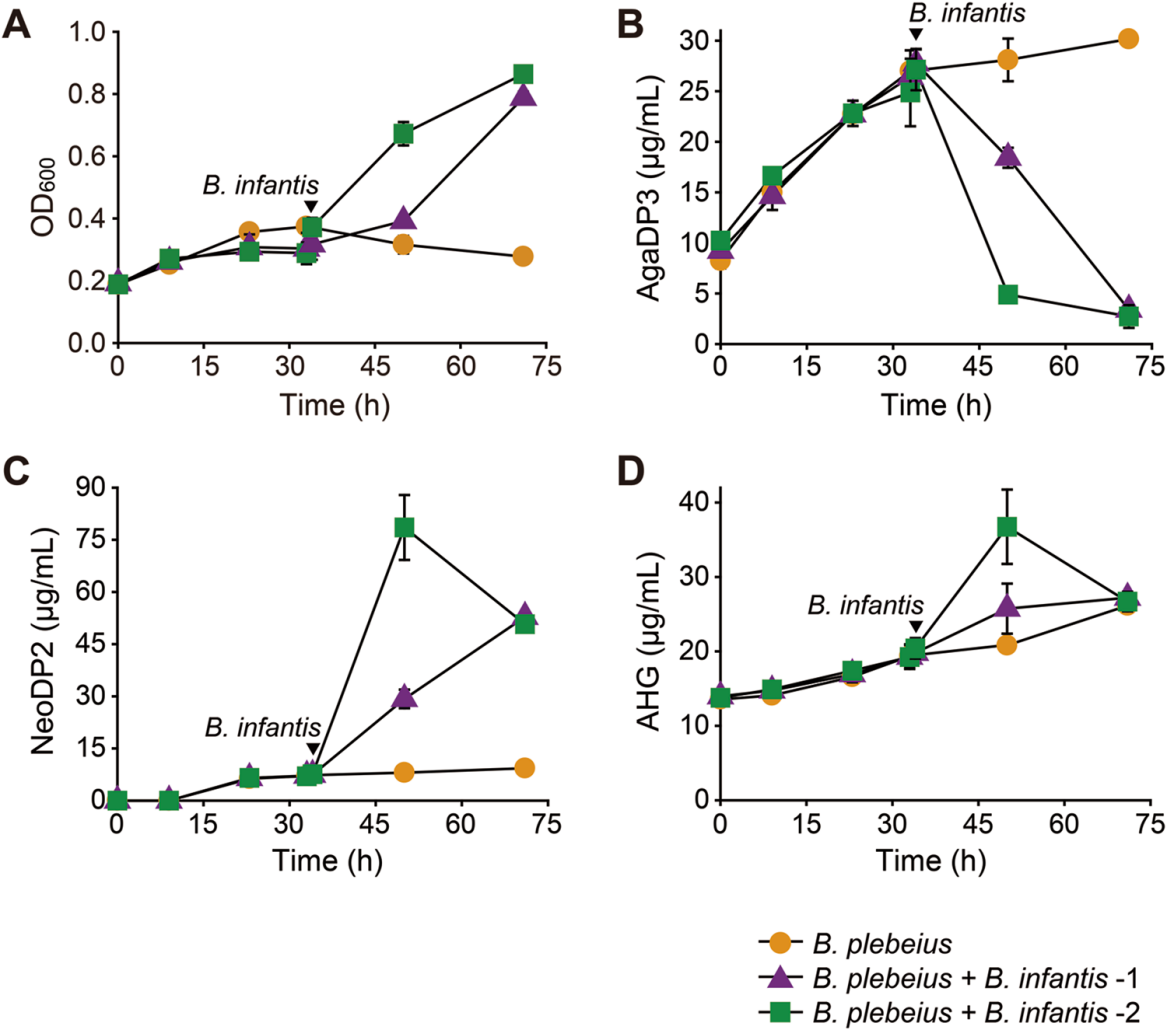
Sequential co-culture of *B. plebeius* (inoculated initially) and *B. infantis* (inoculated later at the two different levels of CFUs) for simulating the cooperative actions of the two distinct gut bacteria with regard to agarose degradation. (**A**) Profiles of cell growth represented by cell density measured at 600 nm and fermentation products, AgaDP3 (**B**), NeoDP2 (**C**), and AHG (**D**), during the fermentation of pretreated agarose with *B. plebeius* only (*B. plebeius* in A − D), *B. plebeius* and *B. infantis* with a low level of inoculum (*B. plebeius* + *B. infantis* – 1 in A − D), or *B. plebeius* and *B. infantis* with a high level of inoculum (*B. plebeius* + *B. infantis* – 2 in A − D). Error bars represent means ± S.D.

During the fermentation of NeoDP2 by *B. plebeius*, AHG was accumulated in the culture supernatant (Supplementary Fig. 7). Therefore, we hypothesized that agarose might be completely hydrolyzed into its monomers, galactose and AHG, through the cooperative actions of the two distinct gut bacteria, *B. plebeius* and *B. infantis*. To prove this, we sequentially co-cultured *B. plebeius* and *B. infantis* with pretreated agarose (Fig. 4A–D). When *B. plebeius* growth reached the stationary phase at 34 h, cells of *B. infantis* were inoculated into the culture broth with an optical density at 600 nm (OD_600_) of 0.013 or 0.083 (corresponding to ~ 1.5 × 10^6^ or ~ 9.2 × 10^6^ colony-forming units (CFU)/mL, respectively)^41^. Agarose degradation intermediates (AgaDP3, NeoDP2, and AHG) were produced during the fermentation of pretreated agarose by *B. plebeius* and *B. infantis* (Supplementary Fig. 8). As already shown in Fig. 1B, during the fermentation of pretreated agarose with *B. plebeius*, AgaDP3 was accumulated in the culture broth (Fig. 4B). Interestingly, after the inoculation with *B. infantis* at 34 h, the concentration of AgaDP3 decreased significantly, and NeoDP2 was produced by the agarolytic β-galactosidase activity of *B. infantis* on AgaDP3 (Fig. 4B,C). The AHG concentration in the culture supernatant was also increased by the cooperative degrading actions of the two distinct bacteria on the pretreated agarose (Fig. 4D). The highest concentration of AHG produced from the sequential co-culture of *B. plebeius* and *B. infantis* was 36.8 mg/L (Fig. 4D). However, after 50 h, a decrease of AHG concentration in the culture of *B. plebeius* + *B. infantis*-2 was observed (Fig. 4D); that is probably due to the conversion of AHG by promiscuous activities of enzymes from *B. plebeius* or *B. infantis*^42^. Also, to determine whether the presence of agarose support cell growth of the co-culture of both bacteria, the growth profiles of the sequential co-culture of *B. plebeius* and then *B. infantis* grown in the presence of agarose or in the absence of agarose were compared but they were almost identical (Supplementary Fig. 9). These results imply that although the agarose-based sugars, AgaDP3, NeoDP2, and AHG, were released by the actions of *B. plebeius* and *B. infantis*, the amounts of those sugars were not sufficient to support their cell growth.

## Discussion

**Figure 5:**
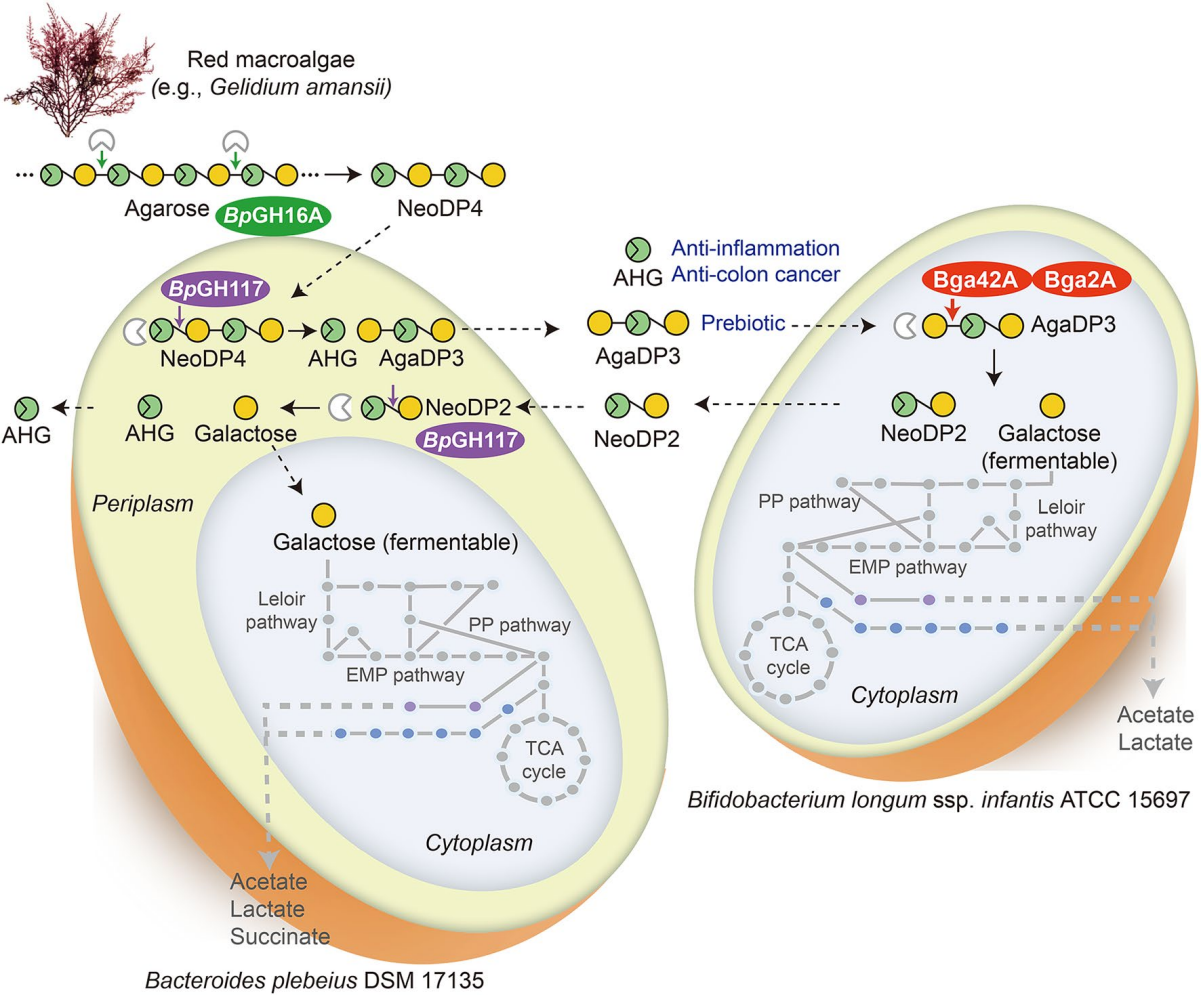
Cooperative actions of two distinct gut bacteria, *B. plebeius* and *B*. *infantis*, enable the complete degradation of seaweed agar. Agarose is initially decomposed into NeoDP4 by *Bp*GH16A, and NeoDP4 is then further hydrolyzed into AgaDP3 and AHG by *Bp*GH117. *B. infantis* hydrolyzes AgaDP3 into NeoDP2 and galactose using Bga42A or Bga2A. Finally, NeoDP2 is hydrolyzed into the monomeric sugars, galactose and AHG by *Bp*GH117.

In this study, we propose a cooperative relationship between two distinct human gut symbionts that results in complementary metabolic activities on agarose, which may explain consuming red seaweed is associated with therapeutic or preventative effects against various diseases and health concerns. First, we characterized the agarose degradation pathway of *B. plebeius*, a microbiome species predominantly observed in East Asians^3,11^. We found that the incomplete agarose degradation by *B. plebeius* leads to the accumulation of oligosaccharides, primarily AgaDP3 (Figs. 1 and 2). Interestingly, the missing link in the agarose degradation pathway of *B. plebeius* is complemented by the actions of agarolytic β-galactosidases of a well-known probiotic strain, *B. infantis* (Fig. 3). Recently, it was demonstrated that in addition to *B. infantis* ATCC 15697, other probiotic *Bifidobacterium* strains assimilating HMOs also have an agarolytic β-galactosidase activity^43^. Second, this unique microbial symbiosis between two distinct gut bacteria, *B. plebeius* and *B. infantis*, enables the production of AHG, a rare sugar possessing various physiological activities^23,43^, through the degradation of seaweed agarose (Fig. 5).

This is the first proposed degradation pathway showing how agarose or AOSs from red seaweeds agar are completely degraded into their monomers (i.e., AHG and galactose) by the unique cooperative actions of two distinct gut bacteria (Fig. 5). In the proposed pathway, first, *Bp*GH16A, probably located at the cell surface, initially decomposes agarose polysaccharide into NeoDP4, and NeoDP4 is then imported into the periplasmic space (Fig. 5). Second, *Bp*GH117, probably located at the periplasm, further degrades NeoDP4 into AHG and AgaDP3 (Fig. 5). AgaDP3 secreted from *B. plebeius* cells is utilized by *B. infantis* via its agarolytic activities of Bga42A and Bga2A to degrade AgaDP3 into galactose and NeoDP2 (Fig. 5). Finally, NeoDP2 secreted from *B. infantis* cells decomposed to AHG and galactose by the action of *Bp*GH117 of *B. infantis* (Fig. 5)*.* In this study, we did not verify which SusCD complex in *B. plebeius* plays a role in importing agar-induced sugars, NeoDP4 and NeoDP2. Considering the general PUL mechanism, BACPLE 01697‒01698 and BACPLE 01704‒01705, which encode SusCD proteins in the PUL of *B. plebeius* (Fig. 2A), are expected to be responsible for importing agar-induced sugars.

*B. infantis* ATCC 15697 is isolated from the intestine of infant (https://www.atcc.org/products/15697) where HMOs are abundant, and it is known as a typical member of the gastrointestinal microbiota of breastfed infants^44^. Furthermore, for the cultivation of *B. infantis* ATCC 15697*,* HMOs such as lacto-*N*-tetraose, lacto-*N*-neotetraose, and 2′-fucosyllactose, are the preferred carbon sources than AgaDP3 in terms of cell growth rate and maximum cell density measured at OD_600_^43,45^; this implies that Bga42A and Bga2A participate in the degradation of HMOs^40^ and AgaDP3, but they favorably degrade HMOs than AgaDP3.

The molecular mechanisms of cooperative degradation of red seaweed agarose by two human gut bacteria, *B. plebeius* and *B. infantis*, demonstrated in this study is one of a few simple but unique examples elucidating the complex interactions in numerous gut microbiota and their effects on host physiology. The cooperative action of the human gut bacteria on red seaweed agarose implies that other polysaccharides that are more complex than agarose may be also degraded by cooperative actions of human gut bacteria in the real conditions of the human gut.

For the verification of this system in vivo using an animal model, porphyran should be considered for supporting the colonization of *B. plebeius* in the gut of animal models; this is because porphyran is a selective nutrient for *B. plebeius* in the competitive gut environment^46,47^. Although agarose is not used as a dietary polysaccharide, agarose is the major component of agar that is widely used for making various types of foods such as jelly and noodles. Therefore, for the future in vivo study using an animal model, food-grade edible agar should be considered along with native porphyran.

## Methods

### Bacterial growth

We cultured *B. plebeius* DSM 17135 (DSMZ, Braunschweig, Germany) in Columbia broth containing 5% (v/v) sheep blood or the minimal broth as previously described^29^ with a slight modification as follows: 4.5 mL of chopped meat carbohydrate broth (Thermo Fisher Scientific, Waltham, MA, USA) or chopped meat glucose broth (Thermo Fisher Scientific), 0.5 mL of trace mineral solution, 0.5 mL of purine and pyrimidine solution, 0.5 mL of vitamin mixture, 0.5 mL of amino acid solution, 0.2 mL of additional nutrient solution, and 0.6 mL of a carbon source such as agarose, AgaDP3, NeoDP2, or glucose, with a final concentration of 5 g/L each.

We cultured *B. infantis* ATCC 15697 in de Man, Rogosa and Sharpe (MRS; Sigma-Aldrich, St. Louis, MO, USA) or synthetic MRS (sMRS) broth^48^. The sMRS broth was composed of 10 g/L peptone, 5 g/L yeast extract, 2 g/L anhydrous dipotassium phosphate, 5 g/L anhydrous sodium acetate, 2 g/L tribasic ammonium citrate, 0.2 g/L magnesium sulfate heptahydrate, 0.05 g/L manganese (II) sulfate, 1 mL/L polysorbate 80, 0.5 g/L cysteine, and 5 g/L carbon source such as AgaDP3.

The *B. plebeius* and *B. infantis* were cultured in a chamber with an anaerobic atmosphere comprising 90% N_2_ and 10% CO_2_, or 90% N_2_, 5% H_2_, and 5% CO_2_ (Airgas, Radnor, PA, USA) at 37 °C. During the fermentation, cell growth was monitored by measuring the optical density at 600 nm.

### Preparation of crude enzymes

To prepare crude enzymes, 5 mL of each cell culture of *B. plebeius* or *B. infantis* grown on culture media supplemented with agarose or AgaDP3, respectively, was centrifuged at 2,880 × *g* and 4 °C for 30 min. The cell pellet and supernatant were then separated to obtain a cell-free crude extract and extracellular crude enzymes, respectively^49^.

The cell pellet was washed with 5 mL of phosphate-buffered saline (PBS; pH 7.4) to obtain the cell-free crude extract. The washed cell pellet obtained by centrifugation at 2,880 × *g* and 4 °C for 30 min was resuspended with 5 mL of 20 mM Tris–HCl buffer (pH 7.0), and the cells were disrupted by sonication for 3 min. The supernatant containing cell-free crude extract was collected by centrifugation at 24,041 × *g* and 4 °C for 30 min. The soluble cell-free crude extract was concentrated using an Amicon ultrafiltration membrane (Millipore, Billerica, MA, USA).

To obtain the extracellular crude enzymes, the culture supernatant was directly concentrated using an Amicon ultrafiltration membrane with a membrane nominal molecular weight limit of 10 kDa (Millipore). The media components in the concentrated crude extracellular enzymes were washed by adding 10 mL of 20 mM Tris–HCl buffer (pH 7.0) followed by centrifugation at 2,880 × *g* and 4 °C for 30 min. This washing step was repeated three times. The protein concentrations of the cell-free crude extract and extracellular crude enzymes were determined using a bicinchoninic acid (BCA) protein assay kit (Thermo Fisher Scientific).

### Cloning, overexpression, and purification of recombinant proteins

The genomic DNAs of *B. plebeius* and *B. infantis* were extracted using a commercial DNA isolation kit (Qiagen, Germantown, MD, USA) for cloning. Three genes from *B. plebeius*—namely BACPLE_01670 (*bpGH16A*), BACPLE_01671 (*bpGH117*), and BACPLE_01683 (*bpGH50*)—and four genes from *B. infantis*—namely Blon_2016 (*bga42A*), Blon_2123 (*bga42B*), Blon_2334 (*bga2A*), and Blon_2416 (*bga42C*)—were amplified from each genomic DNA by polymerase chain reaction (PCR) using the primers described in Table 1. The predicted signal sequences at the *N*-termini of BACPLE_01670, BACPLE_01671, and BACPLE_01671 were removed to facilitate protein expression. Sequentially, the PCR products of each gene and pET21a (Novagen, Madison, WI, USA) were digested with restriction enzymes and ligated using a T4 DNA ligase (Biolabs, Ipswich, MA, USA). The resulting pET21a vector harboring each target gene was then transformed into *Escherichia coli* BL21(DE3) (Novagen, Seoul, Republic of Korea). Strains and plasmids used in this study are listed in Table 2.

**Table 1:** Primers used in this study.

Primer	Sequence (restriction sites are underlined)
F_*Bpgh16A* (*Nde*I)	ATACATATGGCAGAA AATTTA AATAATAAATCATACGAGTG
R_*Bpgh16A* (*Not*I)	AGTGCGGCCGCTTCTTCTGGGACCAGTGATTAAACCC
F_ *Bpgh50* (*Nde*I)	ATACATATGAATACAGGCAATACACAGACTATTGCCG
R_ *Bpgh50* (*Not*I)	AGTGCGGCCGCTTTACGTTCTTTTGATTCACCCTTAGCAG
F_ *Bpgh117* (*Nde*I)	ATACATATGTTGAGTGTTCCGTTCTTCGCTTTGTCATGTG
R_ *Bpgh117* (*Not*I)	AGTGCGGCCGCTTTGTCGAAATAATCTATAAGATTATAAAC
F_ BACPLE_01672 (*Nde*I)	ATACATATGTCTGATTCAAATGTTGATTTCAATAAAGA
R_ BACPLE_01672 (*Xho*I)	AGTCTCGAGTTTTATATTAATTGTTAGTTTATTCGACTG
F_ BACPLE_01673 (*Nde*I)	ATACATATGGGAACCTCCACTAAAGTGGATT
R_ BACPLE_01673 (*Xho*I)	AGTCTCGAGATGTGATATAGGGTGGGAAATCA
F_ BACPLE_01706 (*Nde*I)	ATACATATGATGAACTTGAACTTAAAATCTATTTTTTCAT
R_ BACPLE_01706 (*Xho*I)	AGTCTCGAGATATATTTTTCTGCCCATTTCCTTAAG
F_*bga2A* (*Nde*I)	GGAATTCATATGACAGACGTCACACATGTCGATCGC
R_*bga2A* (*Hind*III)	GAAGCTTGATCAGCTCGAGATCGACGTCGAGG
F_*bga42A* (*Nde*I)	GGAATTCATATGGAACATAGAGCGTTCAAGTGGCCGCA
R_*bga42A* (*Not*I)	GGCGGCCGCCAGCTTGACGACGAGTACGCCGTTG
F_*bga42B* (*Bam*HI)	GGAATTGGATCCATGCGTGCGCGACGTGACTTCGCATG
R_*bga42B* (*Not*I)	GGCGGCCGCCACCGACGGGTTCGGGCGTTTCAT
F_*bga42C* (*Nde*I)	GGAATTCATATGACCGACACCATGGCACACACCCAAC
R_*bga42C* (*Hind*III)	GAAGCTTTGCCGCGGTGCGCACCACCG

**Table 2:** Strains and plasmids used in this study.

Strain or plasmid	Relevant characteristic, genotype, or gene sequence	Source
***Strain***		
*Bacteroides plebeius* DSM 17135	Isolated from feces of Japanese individuals	DSMZ (Braunschweig, Germany)
*Bifidobacterium longum* ssp. *infantis* ATCC 15697	Isolated from feces of human infant	ATCC (Manassas, VA, USA)
*Escherichia coli* DH5α	F– φ80lacZΔ M15 Δ (*lacZYA-argF*) *U169 recA1 endA1 hsdR17* (rK– mK +) *phoA supE44* λ- *thi–1 gyrA96 relA1*	Invitrogen (Carlsbad, CA, USA)
*Escherichia coli* BL21(DE3)	F– *omp*T *hsdS*_*B*_ (r_B_–, m_B_–) *gal dcm* (DE3)	Invitrogen (Carlsbad, CA, USA)
***Plasmid***		
pET21a	T7 promoter with MCS, pBR322 replicon, Amp^R^	Novagen (Burlington, MA, USA)
p*Bpgh16A*	pET21a harboring *Bpgh16A*	This study
p*Bpgh50*	pET21a harboring *Bpgh50*	This study
p*Bpgh117*	pET21a harboring *Bpgh117*	This study
pBACPLE_01672	pET21a harboring BACPLE_01672	This study
pBACPLE_01673	pET21a harboring BACPLE_01673	This study
pBACPLE_01706	pET21a harboring BACPLE_01706	This study
p*bga2A*	pET21a harboring *bga2A*	This study
p*bga42A*	pET21a harboring *bga42A*	This study
p*bga42B*	pET21a harboring *bga42B*	This study
p*bga42C*	pET21a harboring *bga42C*	This study

To overexpress the recombinant proteins, *E. coli* BL21(DE3) harboring each gene was grown at 37 °C in Luria–Bertani broth (Merck, Darmstadt, Germany) containing 100 μg/mL of ampicillin until the mid-exponential phase of growth. Protein expression was then induced by adding 0.1 mM isopropyl-β-d-thiogalactopyranoside (IPTG; Sigma-Aldrich) at 16 °C and further incubating for 16 h. The cells were harvested by centrifugation at 12,857 × *g* for 40 min at 4 °C, and the cell pellet was resuspended in a 20 mM Tris–HCl buffer (pH 7.4) for the purification process.

To purify each of the recombinant proteins, the resuspended cells were disrupted using a sonicator (Branson, Gunpo, Korea), and the supernatant was collected by centrifugation at 24,041 × *g* for 1 h at 4 °C. Each of the recombinant enzymes was purified using a His-Trap column (GE Healthcare, Buckinghamshire, UK). After purification, the recombinant proteins were identified by sodium dodecyl sulfate–polyacrylamide gel electrophoresis (SDS-PAGE) analysis, and protein concentrations were measured using a BCA protein assay kit (Thermo Fisher Scientific).

### In vitro agarase activity

We incubated 100 µL of a reaction mixture comprising 1.5 mg/mL crude enzymes (i.e., cell-free extract or extracellular enzymes) and 0.25% (w/v) agarose in 20 mM Tris–HCl buffer (pH 7.0) at 30 °C and 200 rpm for 12 h to measure the agarase activity of the crude enzymes of *B. plebeius*. The enzymatic reactions were stopped by heating the reaction samples at 95 °C for 2 min.

To measure the agarase activities of *Bp*GH16A and *Bp*GH50, 4 mL of a reaction mixture comprising 0.08 mg/mL (equivalent to 2.32 μM) *Bp*GH16A or 0.06 mg/mL (equivalent to 1.01 μM) *Bp*GH50 and 1% (w/v) agarose or 5 g/L NeoDP4 in 20 mM Tris–HCl buffer (pH 7.0) was incubated at 40 °C and 200 rpm for 6 h. The enzymatic reactions were stopped by heating the reaction samples in boiling water for 3 min. After terminating the enzymatic reactions, the reaction products from each sample were identified by LC/MS − IT − TOF, TLC, or high-performance liquid chromatography (HPLC) analyses.

### In vitro NABH activity

We incubated 100 µL of a reaction mixture comprising 1.5 mg/mL crude enzymes (i.e., cell-free extract or extracellular enzymes) and 2.5 mg/mL NeoDP2 in 20 mM Tris–HCl buffer (pH 7.0) at 30 °C and 200 rpm for 12 h to measure the NABH activity of the crude enzymes of *B. plebeius*. The enzyme reactions were stopped by heating the reaction samples at 95 °C for 2 min.

To measure the NABH activity of *Bp*GH117, 4 mL of a reaction mixture comprising 0.02 mg/mL (equivalent to 0.46 μM) *Bp*GH117 and 5 mg/mL NeoDP2 in 20 mM Tris–HCl buffer (pH 7.0) was incubated at 40 °C and 200 rpm for 2 h. The enzymatic reaction was stopped by heating the reaction sample in boiling water for 3 min. After terminating the enzymatic reactions, the reaction products from each sample were identified by TLC and HPLC analyses.

### Sequential or simultaneous enzymatic hydrolysis of agarose

For the sequential enzymatic reactions of *Bp*GH16A and *Bp*GH117, we first incubated a reaction mixture comprising 0.08 mg/mL (equivalent to 2.32 μM) *Bp*GH16A and 1% (w/v) agarose in 10 mM Tris–HCl buffer (pH 7.0) at 40 °C and 200 rpm for 6 h. After inactivation of the first enzymatic reaction by heating the reaction sample at 95 °C for 3 min, *Bp*GH117 was added to the reaction products of *Bp*GH16A with a final concentration of 0.08 mg/mL (equivalent to 1.83 μM), and the reaction mixture was incubated at 40 °C and 200 rpm for 2 h. The final substrate loading was diluted twofold in the second reaction step.

For the simultaneous enzymatic reaction of *Bp*GH16A and *Bp*GH117, we incubated a reaction mixture comprising *Bp*GH16A and *Bp*GH117 with a final concentration of 0.08 mg/mL (equivalent to 2.32 and 1.83 μM each, respectively) and 1% (w/v) agarose in 10 mM Tris–HCl buffer (pH 7.0) at 40 °C for 6 h. The enzymatic reactions were then terminated by heating the reaction sample in boiling water for 3 min. After the sequential or simultaneous enzymatic hydrolyses of agarose by *Bp*GH16A and *Bp*GH117, the reaction products were identified by TLC.

### Effect of simulated gastric fluid on agarose hydrolysis

To investigate the effect of simulated gastric fluid on agarose hydrolysis, we incubated 1% (w/v) agarose in a simulated gastric fluid comprising 0.2% (w/v) sodium chloride in 0.7% (v/v) hydrochloric acid (pH 1.11) at 37 °C for 2 h. The reaction sample was then neutralized to pH 7.0, which is the pH of the distal colon, using 1 M NaOH solution, and pre-digested agarose was incubated with *Bp*GH16A and *Bp*GH117 either sequentially or simultaneously.

As described above, *Bp*GH16A with a final concentration of 0.08 mg/mL was incubated with 1% (w/v) pre-digested agarose using a simulated gastric fluid as a substrate at 37 °C for 2 h. After terminating the first reaction by heating the reaction sample in boiling water for 3 min, *Bp*GH117 with a final concentration of 0.08 mg/mL was sequentially incubated with the first reaction products at 37 °C for 2 h. The second reaction was terminated by heating the reaction sample in boiling water for 3 min. *Bp*GH16A and *Bp*GH117 with final concentrations of 0.08 mg/mL each were incubated simultaneously with 1% (w/v) pre-digested agarose using a simulated gastric fluid at 37 °C for 2 h.

### Assay of agarolytic β-galactosidase activity

To determine the enzyme activities of the β-galactosidases from *B. infantis* ATCC 15697—i.e., Bga42A, Bga42B, Bga2A, and Bga42C—we incubated 1 nmol of each β-galactosidase in 100 µL of 50 mM sodium phosphate buffer (pH 6.0) containing 5 mM AgaDP3 and 1 mM MgCl_2_ at 30 °C and 200 rpm for 2 h. After terminating the enzymatic reactions by boiling the reaction samples for 3 min, we determined the amount of galactose by HPLC. One unit of agarolytic β-galactosidase activity with regard to AgaDP3 was defined as the amount of enzyme required to produce 1 µmol of galactose per min under the reaction conditions described above. To investigate the crude enzyme reactions of *B. infantis*, we incubated a reaction mixture comprising the 2 mg/mL crude enzymes (i.e., cell-free extract or extracellular enzymes) and 2 mg/mL AgaDP3 in 20 mM Tris HCl buffer (pH 7.0) at 30 °C and 200 rpm for 2 h. We used lactose as a positive control substrate for determining β-galactosidase activity. The agarolytic β-galactosidase activities of the crude enzymes and the recombinant enzymes belonging to GH2 from *B. plebeius*, the recombinant enzymes of BACPLE_01672, BACPLE_01673, and BACPLE_01706, were tested using the same method described above.

### LC/MS − IT − TOF analysis

We used an LC/MS − IT − TOF system (Shimadzu, Kyoto, Japan) equipped with a Hypercarb Porous Graphitic Carbon LC column (100 mm × 2.1 mm, packed with 3-µm particles; Thermo Fisher Scientific) to analyze the NAOSs and AOSs derived from the agarose^50^. During the analysis, the temperatures of the autosampler and LC column were maintained at 10 °C and 70 °C, respectively. The mobile phase comprised two solutions—25 µM lithium chloride and acetonitrile—for gradient elution ranging from 0 to 80% at a flow rate of 0.2 mL/min for 41 min. Electrospray ionization was operated in a positive-ion mode. The source-dependent parameters were set as follows: nebulizing gas flow, 1.5 L/min; interface voltage, 4.5 kV; detector voltage, 1.65 kV; curved desolvation line temperature, 200 °C; and heat block temperature, 200 °C. The mass scan range was 100–1200 *m﻿/z*. To obtain tandem mass data from the precursor ions, the collision-induced dissociation (CID) parameters—i.e., the CID energy, collision gas parameter, and frequency—were set at 50%, 50%, and 45 kHz, respectively. The raw data obtained from the LC/MS − IT − TOF analysis were processed using LabSolutions LCMS software (version 3.8; Shimadzu).

### Gas chromatography − mass spectrometry analysis

To analyze AHG produced from cell culture or enzymatic reaction with agarose, gas chromatography − mass spectrometry (GC − MS) was used. The culture supernatant or reaction product was centrifuged at 25,200 × *g* for 10 min at 4 °C, and 10 µL of the supernatant was dried in a centrifugal vacuum evaporator. For chemical derivatization, we added 40 mg/mL methoxyamine hydrochloride in pyridine (Sigma-Aldrich) to the vacuum-dried sample, and incubated the mixture for 90 min at 30 °C. We then mixed each sample with 45 µL of *N*-methyl-*N*-(trimethylsilyl)-trifluoroacetamide (MSTFA; Sigma-Aldrich), and incubated the mixture for 30 min at 37 °C. The derivatized samples were analyzed using an Agilent 7890A GC/5975C MSD system (Agilent Technologies, Wilmington, DE, USA) equipped with an RTX-5Sil MS column (30 m × 0.25 mm; film thickness, 0.25 μm) (Restek, Bellefonte, PA, USA) with a 10-m guard column. Each sample (1 µL) was injected into the GC using the split mode with a split ratio of 2. The oven program was as follows: an initial temperature of 50 °C for 1 min; heating to 330 °C at 20 °C/min; and maintenance of the temperature at 330 °C for 5 min. Electron ionization was performed at 70 eV, and the temperatures of the ion source and transfer line were 250 °C and 280 °C, respectively. The mass spectra of the samples were obtained in the mass range 85–500 *m﻿/z﻿*.

For the quantitative analyses of AgaDP3, NeoDP2, and AHG, an Agilent 7890A GC (Agilent Technologies) coupled with a Pegasus HT time-of-flight (TOF) − mass spectrometry (MS) (LECO, St. Joseph, MI) was used. Each sample (1 µL) was injected into the GC using the split mode with a split ratio of 2 and separated on an RTX-5Sil MS column (30 m × 0.25 mm; film thickness, 0.25 μm) (Restek, Bellefonte, PA, USA) with a 10-m guard column. The oven program was as follows: an initial temperature of 50 °C for 1 min; heating to 330 °C at 20 °C/min; and maintenance of the temperature at 330 °C for 5 min. Electron ionization was performed at 70 eV, and the temperatures of the ion source and transfer line were 250 °C and 280 °C, respectively. The mass spectra of the samples were obtained in the mass range 85–500 *﻿m/z*.

### HPLC analysis

We used a HPLC system (1200 Series, Agilent Technologies) equipped with a H^+^ (8%) column (Rezex ROA-Organic Acid; Phenomenex, Torrance, CA, USA) and a refractive index (RI) detector for the HPLC analysis. The flow rate of the 0.005 N H_2_SO_4_ mobile phase was set at 0.6 mL/min, and the column and refractive index detector temperatures were set at 50 °C.

### TLC analysis

TLC analysis of the enzymatic reaction products was conducted on a silica gel 60 plate (Merck), and the plate was developed with an *n*-butanol–ethanol–water mixture (3:1:1, v/v/v) for 1 h. The plate was then dried and visualized using a solution comprising 10% (v/v) sulfuric acid and 0.2% (w/v) 1,3-dihydroxynaphthalene (Sigma-Aldrich) in ethanol at 90 °C for 1 min.

### Accession numbers of recombinant enzymes

The NCBI accession numbers of the proteins analyzed in this study are the following: *Bp*GH16A, WP_007560915.1; *Bp*GH50, WP_007560940.1; *Bp*GH117, WP_007560917.1; Bga2A, WP_012578561.1; Bga42A, WP_012578289.1; Bga42B, WP_012578388.1; Bga42C, WP_012578637.1.

## Supplementary Information

Supplementary Information.

## Data Availability

All data are available in the main text or the additional information.

## References

[1] Ndeh D (2017). Complex pectin metabolism by gut bacteria reveals novel catalytic functions. Nature.

[2] Luis AS (2018). Dietary pectic glycans are degraded by coordinated enzyme pathways in human colonic Bacteroides. Nat. Microbiol..

[3] Hehemann J-H (2010). Transfer of carbohydrate-active enzymes from marine bacteria to Japanese gut microbiota. Nature.

[4] Flint HJ, Scott KP, Duncan SH, Louis P, Forano E (2012). Microbial degradation of complex carbohydrates in the gut. Gut Microbes.

[5] Ley RE, Turnbaugh PJ, Klein S, Gordon JI (2006). Microbial ecology: human gut microbes associated with obesity. Nature.

[6] Martens EC, Chiang HC, Gordon JI (2008). Mucosal glycan foraging enhances fitness and transmission of a saccharolytic human gut bacterial symbiont. Cell Host Microbe.

[7] Pickard JM (2014). Rapid fucosylation of intestinal epithelium sustains host-commensal symbiosis in sickness. Nature.

[8] Sonnenburg ED (2010). Specificity of polysaccharide use in intestinal Bacteroides species determines diet-induced microbiota alterations. Cell.

[9] D'Elia JN, Salyers AA (1996). Effect of regulatory protein levels on utilization of starch by *Bacteroides thetaiotaomicron*. J. Bacteriol..

[10] Brune A (2014). Symbiotic digestion of lignocellulose in termite guts. Nat. Rev. Microbiol..

[11] Pluvinage B (2018). Molecular basis of an agarose metabolic pathway acquired by a human intestinal symbiont. Nat. Commun..

[12] Thomas F (2012). Characterization of the first alginolytic operons in a marine bacterium: from their emergence in marine *Flavobacteriia* to their independent transfers to marine *Proteobacteria* and human gut *Bacteroides*. Environ. Microbiol..

[13] Yasui, A. Outline of standard tables of food composition in Japan-2015-(7th revised edition). Vol. 74 (2016).

[14] Lee HJ, Kim HC, Vitek L, Nam CM (2010). Algae consumption and risk of type 2 diabetes: Korean National Health and Nutrition Examination Survey in 2005. J. Nutr. Sci. Vitaminol..

[15] Okubo H (2008). Three major dietary patterns are all independently related to the risk of obesity among 3760 Japanese women aged 18–20 years. Int. J. Obes..

[16] Shimazu T (2007). Dietary patterns and cardiovascular disease mortality in Japan: a prospective cohort study. Int. J. Epidemiol..

[17] Wong KH, Sam SW, Cheung PCK, Ang PO (1999). Changes in lipid profiles of rats fed with seaweed-based diets. Nutr. Res..

[18] Kim N-J, Li H, Jung K, Chang HN, Lee PC (2011). Ethanol production from marine algal hydrolysates using *Escherichia coli* KO11. Bioresour. Technol..

[19] Jang S-S, Shirai Y, Uchida M, Wakisaka M (2012). Production of mono sugar from acid hydrolysis of seaweed. Afr. J. Biotechnol..

[20] Ale MT, Maruyama H, Tamauchi H, Mikkelsen JD, Meyer AS (2011). Fucoidan from *Sargassum* sp. and *Fucus vesiculosus* reduces cell viability of lung carcinoma and melanoma cells *in vitro* and activates natural killer cells in mice *in vivo*. Int. J. Biol. Macromol..

[21] Aisa Y (2005). Fucoidan induces apoptosis of human HS-sultan cells accompanied by activation of caspase-3 and down-regulation of ERK Pathways. Am. J. Hematol..

[22] Kim EJ, Park SY, Lee JY, Park JH (2010). Fucoidan present in brown algae induces apoptosis of human colon cancer cells. BMC Gastroenterol..

[23] Zhang Z, Teruya K, Eto H, Shirahata S (2013). Induction of apoptosis by low-molecular-weight fucoidan through calcium- and caspase-dependent mitochondrial pathways in MDA-MB-231 breast cancer cells. Biosci. Biotechnol. Biochem..

[24] Yun EJ (2013). Enzymatic production of 3,6-anhydro-L-galactose from agarose and its purification and *in vitro* skin whitening and anti-inflammatory activities. Appl. Microbiol. Biotechnol..

[25] Hoshiyama Y, Sekine T, Sasaba T (1993). A case-control study of colorectal cancer and its relation to diet, cigarettes, and alcohol consumption in Saitama Prefecture Japan. Tohoku J. Exp. Med..

[26] Hoshiyama Y, Sasaba T (1992). A case-control study of stomach cancer and its relation to diet, cigarettes, and alcohol consumption in Saitama Prefecture Japan. Cancer Causes Control.

[27] Kato I (1990). A comparative case-control study of colorectal cancer and adenoma. Jpn. J. Cancer Res..

[28] Knutsen S, Myslabodski D, Larsen B, Usov AI (1994). A modified system of nomenclature for red algal galactans. Bot. Mar..

[29] Hehemann J-H, Kelly AG, Pudlo NA, Martens EC, Boraston AB (2012). Bacteria of the human gut microbiome catabolize red seaweed glycans with carbohydrate-active enzyme updates from extrinsic microbes. Proc. Natl. Acad. Sci. U.S.A..

[30] Duckworth M, Turvey JR (1969). The action of a bacterial agarase on agarose, porphyran and alkali-treated porphyran. Biochem. J..

[31] Hehemann J-H, Smyth L, Yadav A, Vocadlo DJ, Boraston AB (2012). Analysis of keystone enzyme in agar hydrolysis provides insight into the degradation (of a polysaccharide from) red seaweeds. J. Biol. Chem..

[32] Park NJ, Yu S, Kim DH, Yun EJ, Kim KH (2021). Characterization of *Bp*GH16A of *Bacteroides plebeius*, a key enzyme initiating the depolymerization of agarose in the human gut. Appl. Microbiol. Biotechnol..

[33] Giles K, Pluvinage B, Boraston AB (2017). Structure of a glycoside hydrolase family 50 enzyme from a subfamily that is enriched in human gut microbiome bacteroidetes. Proteins.

[34] Yang B (2009). Mechanism of mild acid hydrolysis of galactan polysaccharides with highly ordered disaccharide repeats leading to a complete series of exclusively odd-numbered oligosaccharides. FEBS J..

[35] Higashimura Y (2016). Protective effect of agaro-oligosaccharides on gut dysbiosis and colon tumorigenesis in high-fat diet-fed mice. Am. J. Physiol. Gastrointest. Liver Physiol..

[36] Hu B (2006). Prebiotic effects of neoagaro-oligosaccharides prepared by enzymatic hydrolysis of agarose. Anaerobe.

[37] Li MM (2014). Isolation and characterization of an agaro-oligosaccharide (AO)-hydrolyzing bacterium from the gut microflora of Chinese individuals. PLoS ONE.

[38] Garrido D (2013). Utilization of galactooligosaccharides by Bifidobacterium longum subsp infantis isolates. Food Microbiol..

[39] Lee CH (2014). A novel agarolytic β-galactosidase acts on agarooligosaccharides for complete hydrolysis of agarose into monomers. Appl. Environ. Microbiol..

[40] Yoshida E (2012). Bifidobacterium longum subsp infantis uses two different β-galactosidases for selectively degrading type-1 and type-2 human milk oligosaccharides. Glycobiol..

[41] Kavanaugh DW (2013). Exposure of Bifidobacterium longum subsp infantis to milk oligosaccharides increases adhesion to epithelial cells and induces a substantial transcriptional response. PLoS ONE.

[42] Yun EJ (2018). Promiscuous activities of heterologous enzymes lead to unintended metabolic rerouting in *Saccharomyces cerevisiae* engineered to assimilate various sugars from renewable biomass. Biotechnol. Biofuels.

[43] Yun EJ (2021). *In vitro* prebiotic and anti-colon cancer activities of agar-derived sugars from red seaweeds. Mar. Drugs.

[44] Turroni F (2012). Diversity of bifidobacteria within the infant gut microbiota. PLoS ONE.

[45] Garrido D (2016). A novel gene cluster allows preferential utilization of fucosylated milk oligosaccharides in Bifidobacterium longum subsp longum SC596. Sci. Rep..

[46] Shepherd ES, DeLoache WC, Pruss KM, Whitaker WR, Sonnenburg JL (2018). An exclusive metabolic niche enables strain engraftment in the gut microbiota. Nature.

[47] Kearney SM, Gibbons SM, Erdman SE, Alm EJ (2018). Orthogonal dietary niche enables reversible engraftment of a gut bacterial commensal. Cell Rep..

[48] Barrangou R, Altermann E, Hutkins R, Cano R, Klaenhammer TR (2003). Functional and comparative genomic analyses of an operon involved in fructooligosaccharide utilization by *Lactobacillus acidophilus*. Proc. Natl. Acad. Sci. U.S.A..

[49] Yun EJ (2011). Production of 3,6-anhydro-L-galactose from agarose by agarolytic enzymes of *Saccharophagus degradans* 2–40. Process Biochem..

[50] Kim JH, Yun EJ, Yu S, Kim KH, Kang NJ (2017). Different levels of skin whitening activity among 3,6-anhydro-L-galactose, agarooligosaccharides, and neoagarooligosaccharides. Mar. drugs.

