# Supplementary material for: Metabolic and enzymatic elucidation of cooperative degradation of red seaweed agarose by two human gut bacteria
Source: Sci Rep. 2021 Jul 6;11:13955. doi: 10.1038/s41598-021-92872-y (PMC8260779; doi:10.1038/s41598-021-92872-y)
Supplement: Supplementary file 1 — Supplementary Information. [file 41598_2021_92872_MOESM1_ESM.docx]

**Supplementary Information**

**Metabolic and enzymatic elucidation of cooperative degradation of red seaweed agarose by two human gut bacteria**

Eun Ju Yun^1,2^, Sora Yu^1^, Na Jung Park^1^, Yoonho Cho^1^, Na Ree Han^1^, Yong-Su Jin^2,3#^ & Kyoung Heon Kim^1#^

^1^Department of Biotechnology, Graduate School, Korea University, Seoul 02841, Republic of Korea

^2^Carl R. Woese Institute for Genomic Biology, University of Illinois at Urbana-Champaign, Urbana, Illinois 61801, USA

^3^Department of Food Science and Human Nutrition, University of Illinois at Urbana-Champaign, Urbana, Illinois 61801, USA

^#^Address correspondence to khekim@korea.ac.kr (K.H.K.) and ysjin@illinois.edu (Y.S.J.)

**
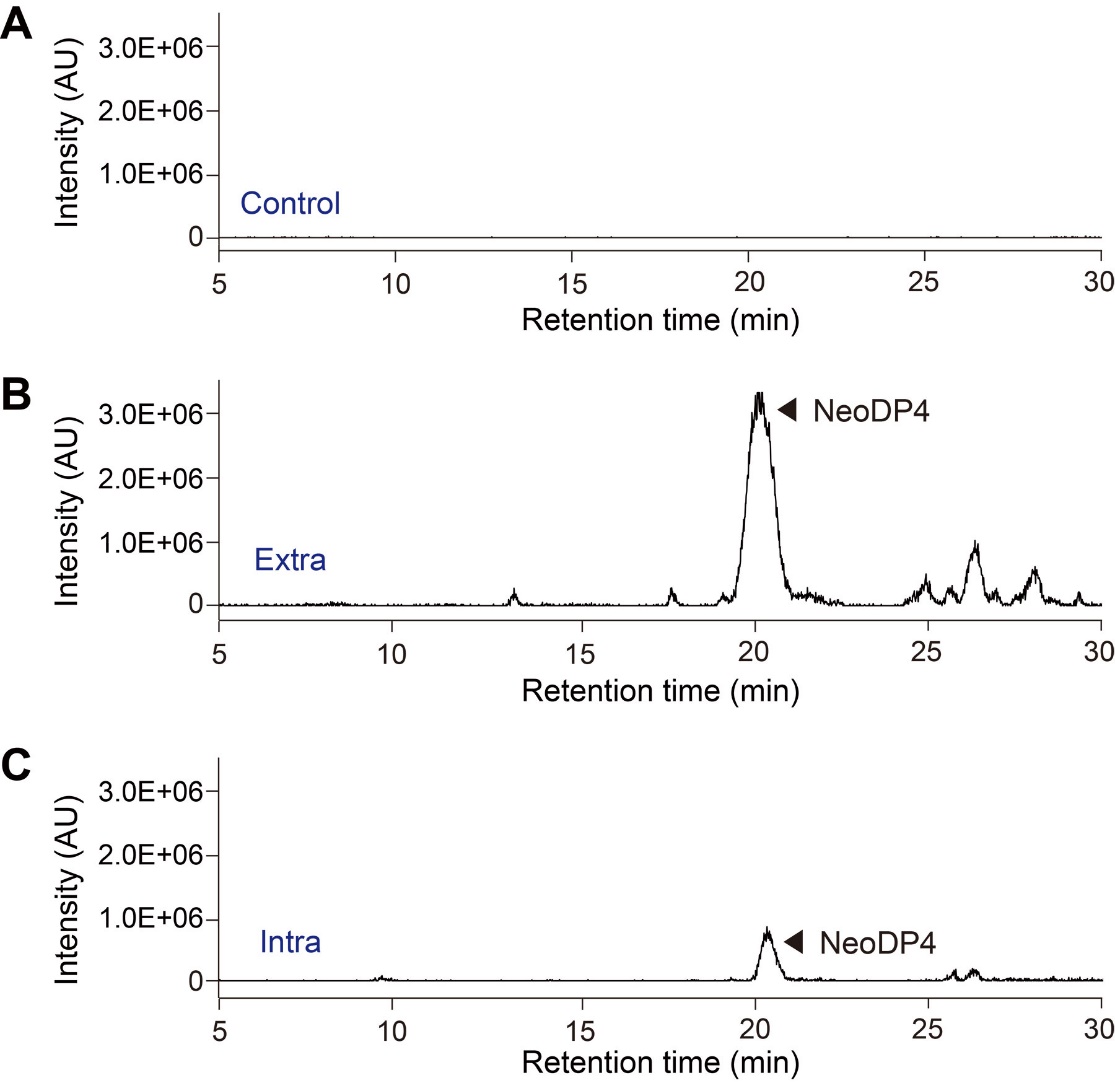
**

**Figure S1.** *In vitro* agarase activities of the crude enzymes of *B. plebeius*. The reaction products resulting from the action of the crude enzymes of *B. plebeius* on agarose were analyzed by LC/MS−IT−TOF. **A**, The reaction mixture without the crude enzymes. **B**, the reaction products of the extracellular crude enzymes with agarose. **C**, The reaction products of the cell-free crude extract with agarose.


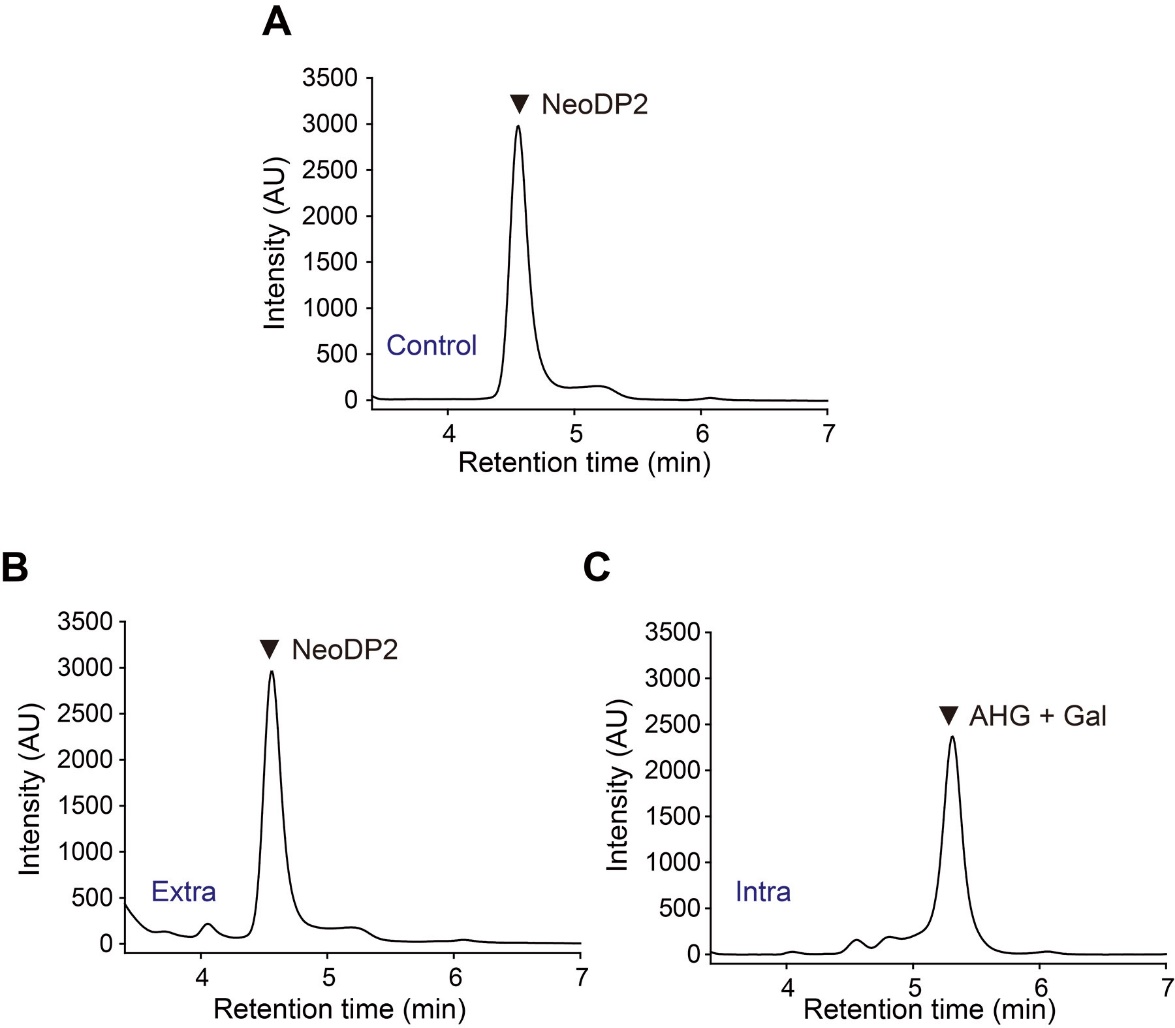


**Figure S2.** *In vitro* NABH activities of the crude enzymes of *B. plebeius***.** The enzymatic reaction products of crude enzymes of *B. plebeius* with NeoDP2 were analyzed by HPLC. **A**, The reaction mixture without the crude enzymes. **B**, The reaction products of the extracellular crude enzymes with NeoDP2. **C**, The reaction products of the cell-free crude extract with NeoDP2.


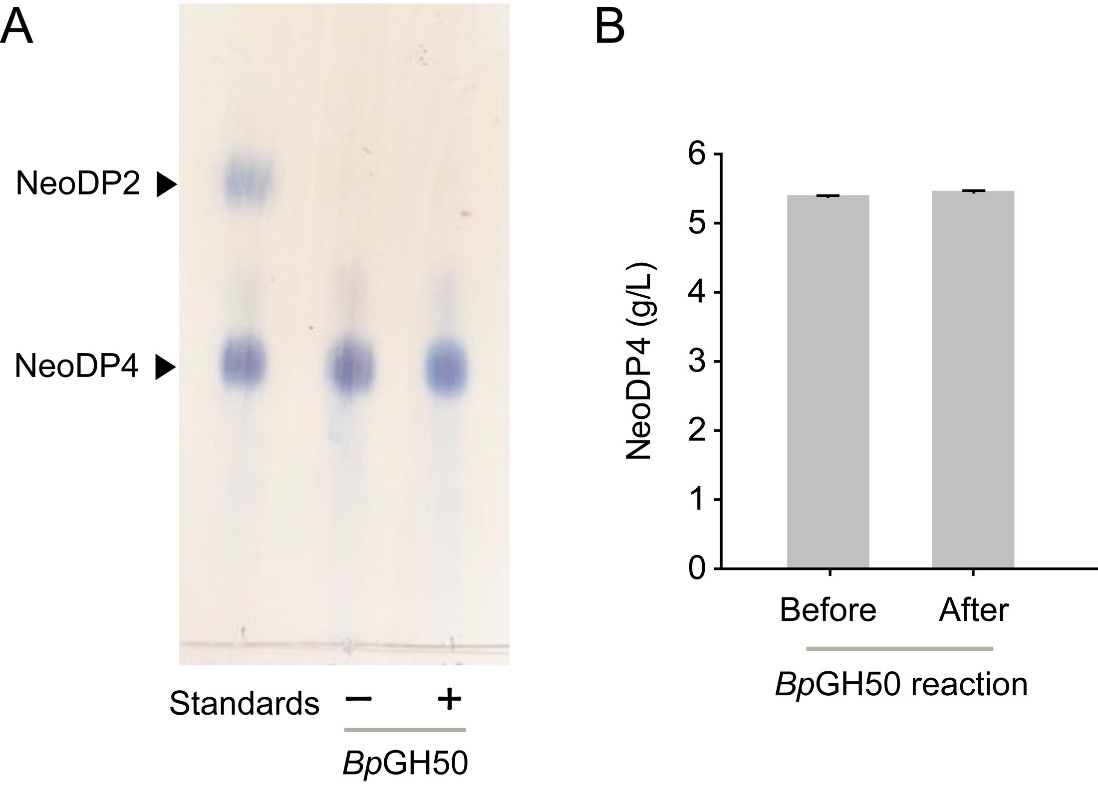


**Figure S3.** The activity of *Bp*GH50 on NeoDP4, a major product produced by *Bp*GH16A reaction with agarose. The activity of *Bp*GH50 was verified by (**A**) TLC and (**B**) HPLC analyses.


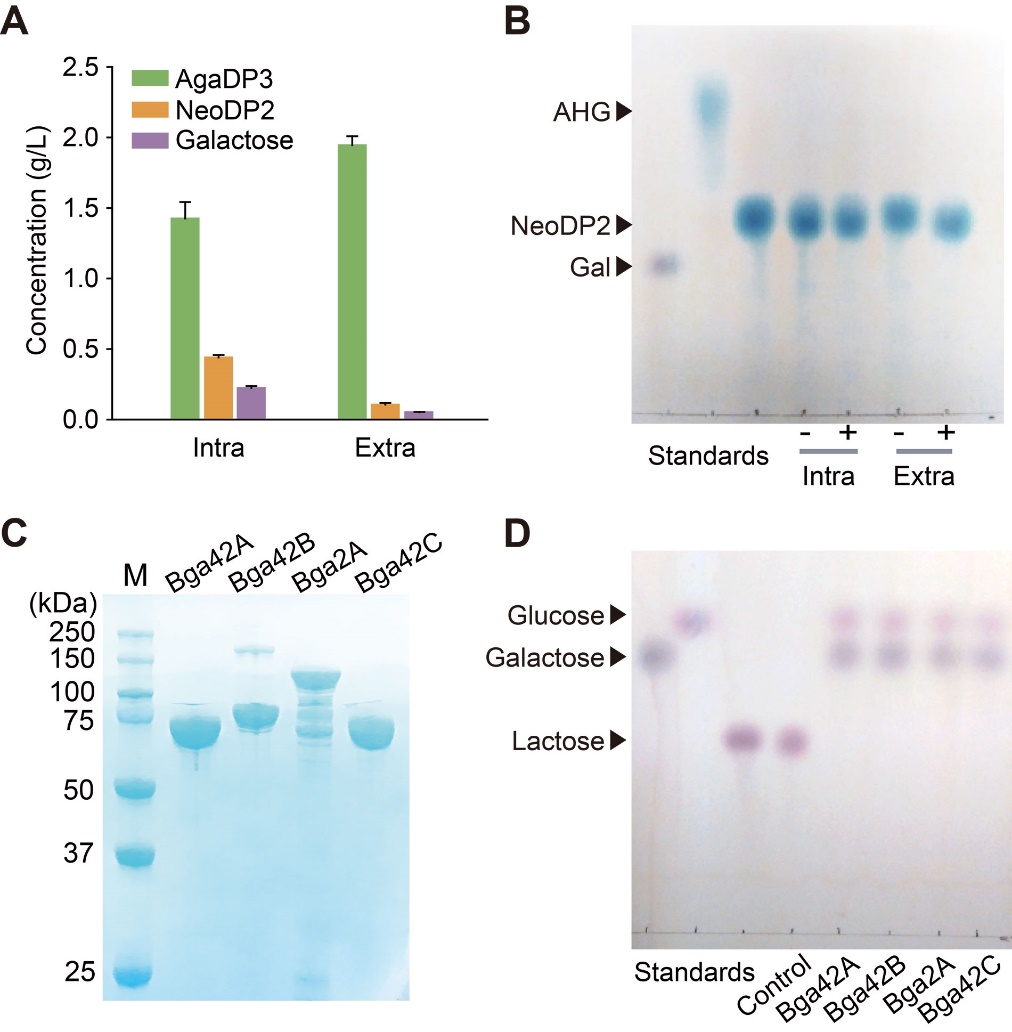


**Figure S4.** *In vitro* β-galactosidase activities of the recombinant β-galactosidases Bga42A, Bga42B, Bga2A, and Bga42C on lactose. To confirm the *in vitro* enzymatic activities, the reaction products were analyzed by TLC.


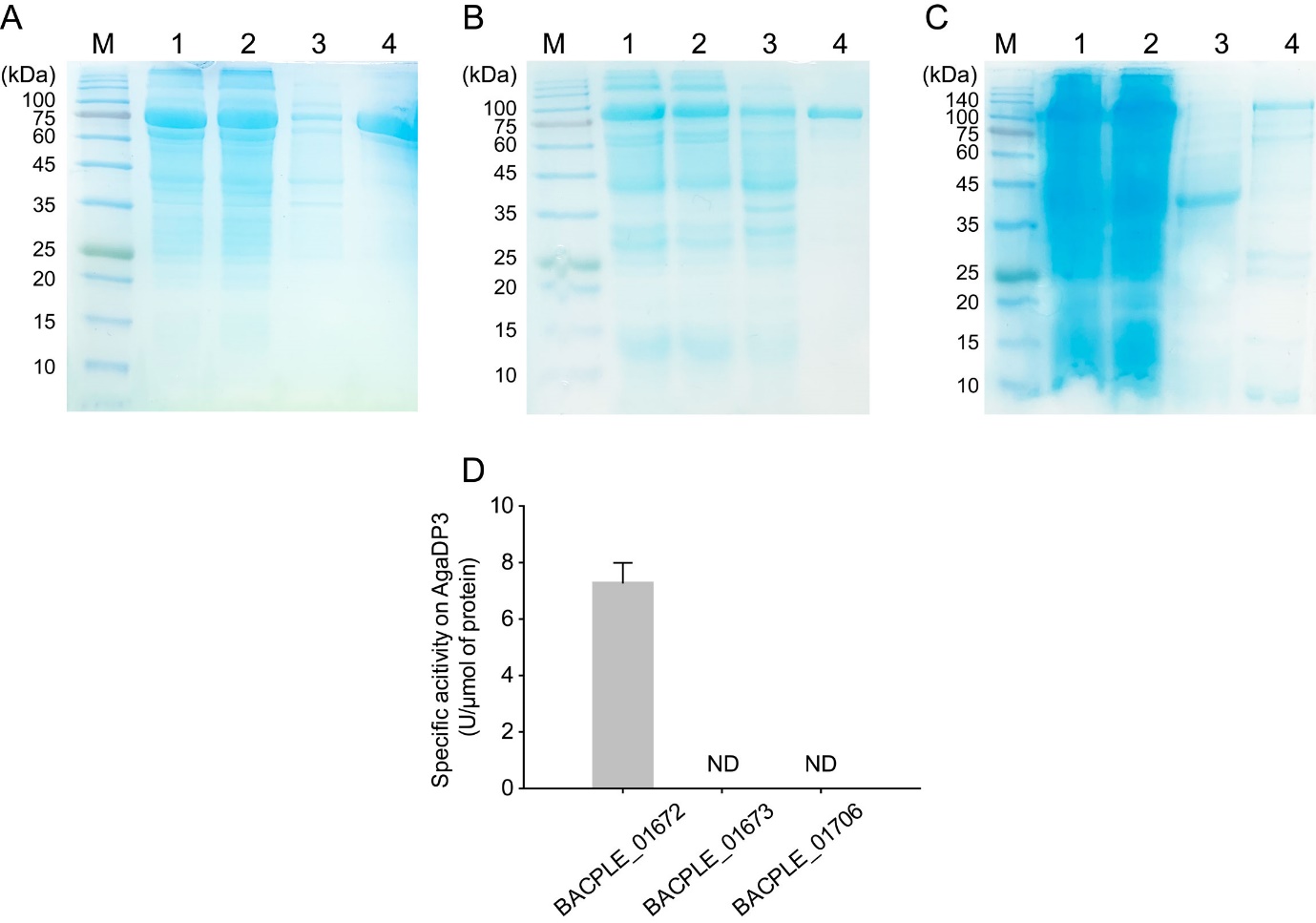


**Figure S5.** Comparison of the specific agarolytic β-galactosidase activities of the recombinant enzymes belonging to GH2 located in the PUL of B. plebeius. (**A**‒**C**) SDS-PAGE analysis of the overexpressed and purified recombinant enzymes of BACPLE_01672 (**A**), BACPLE_01673 (**B**), and BACPLE_01706 (**C**) from B. plebeius. Lanes: M, protein markers; 1, crude extract; 2, flow-through fraction from crude extract loaded into a His-Trap column; 3, wash fraction from washing the His-Trap column using equilibrium buffer; 4‒6, elution fractions from the His-Trap column. **D**. Comparison of the specific agarolytic β-galactosidase activities of the recombinant enzymes of BACPLE_01672, BACPLE_01673, and BACPLE_01706 on AgaDP3. Purification of the agarose degradation pathway enzymes BpGH16A, BpGH50, and BpGH117 originating from B. plebeius. Error bars represent means ± S.D.


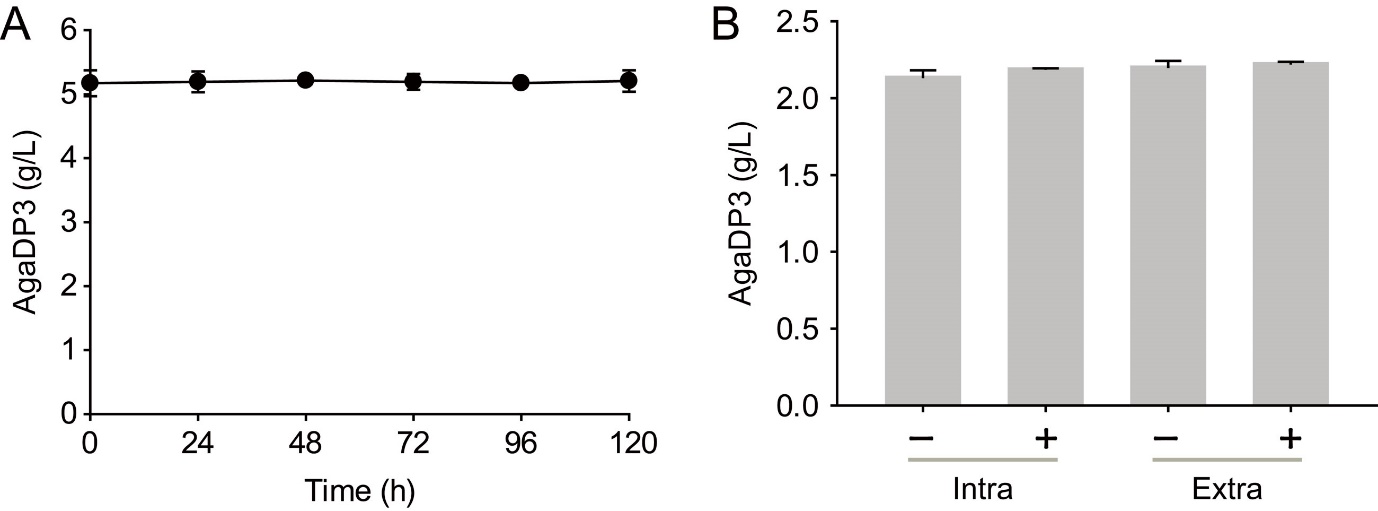


**Figure S6.** Monitoring of an agarolytic β-galactosidase activity of *B. plebeius*. (**A**) Monitoring of the concentration of AgaDP3 in culture supernatant during the fermentation of *B. plebeius*. The cells of B. plebeius were cultured with 5 g/L AgaDP3 for 120 h with an initial OD_600_ at 0.2. (**B**) In vitro agarolytic β-galactosidase activities of the cell-free lysate (Intra) and extracellular crude enzymes (Extra) obtained from B. plebeius. Error bars represent means ± S.D.

**
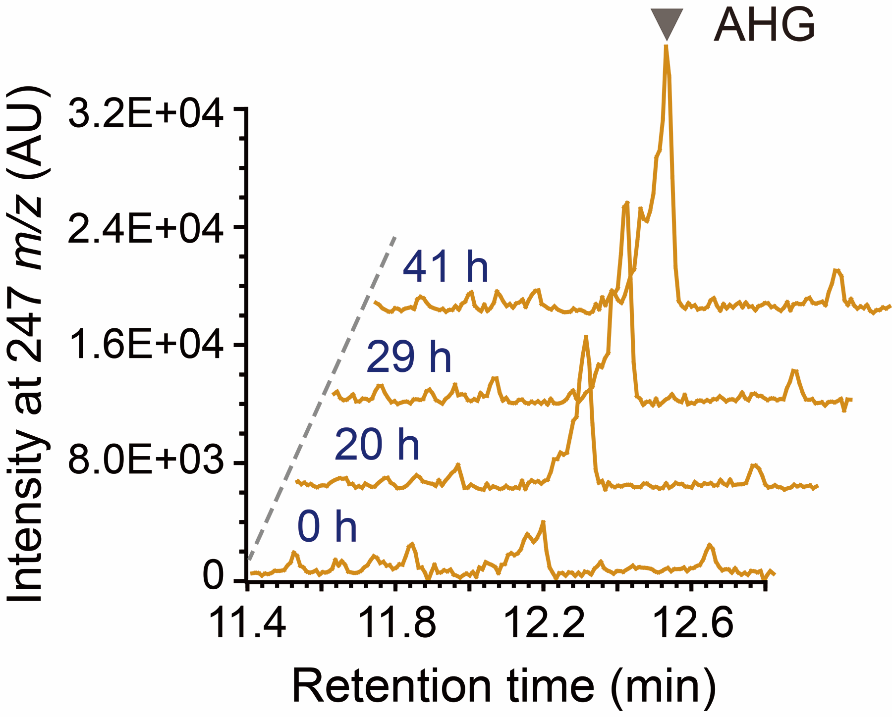
**

**Figure S7.** Abundance change of AHG with time in the culture supernatant during the NeoDP2 fermentation by *B. plebeius.* The peak intensity of AHG was obtained by GC‒MS at 247 *m/z*.

**
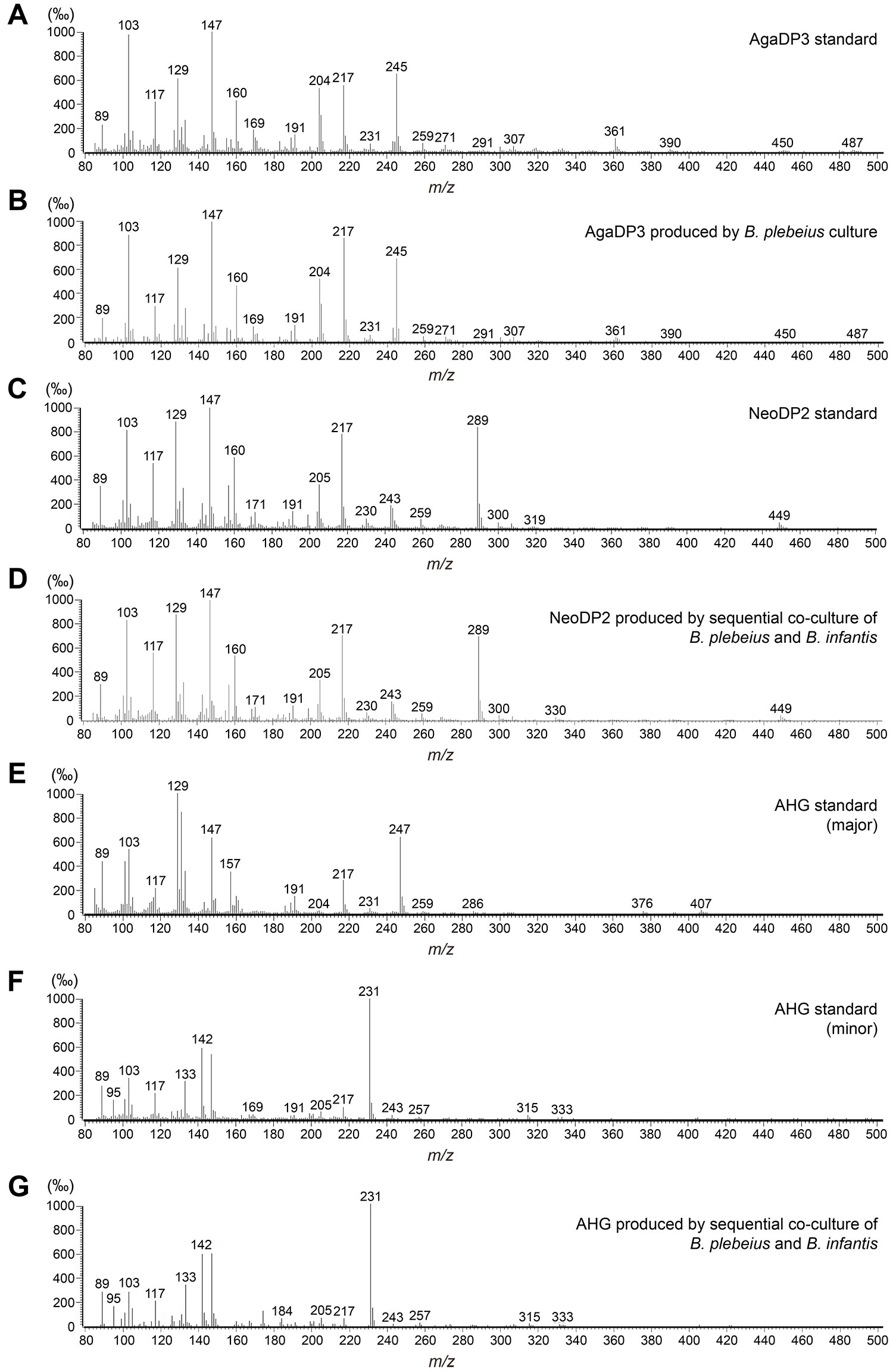
**

**Figure S8.** Mass spectra obtained by GC/TOF MS analyses of AgaDP3, NeoDP2, and AHG. **A**, AgaDP3 standard. **B**, AgaDP3 produced by *B. plebeius* cultured with pretreated agarose. **C**, NeoDP2 standard. **D**, NeoDP2 produced from the sequential co-culture of *B. plebeius* and *B. infantis* with pretreated agarose. **E**, AHG standard (major form). **F**, AHG standard (minor form). **G**, AHG produced from the sequential co-culture of *B. plebeius* and *B. infantis* with pretreated agarose.


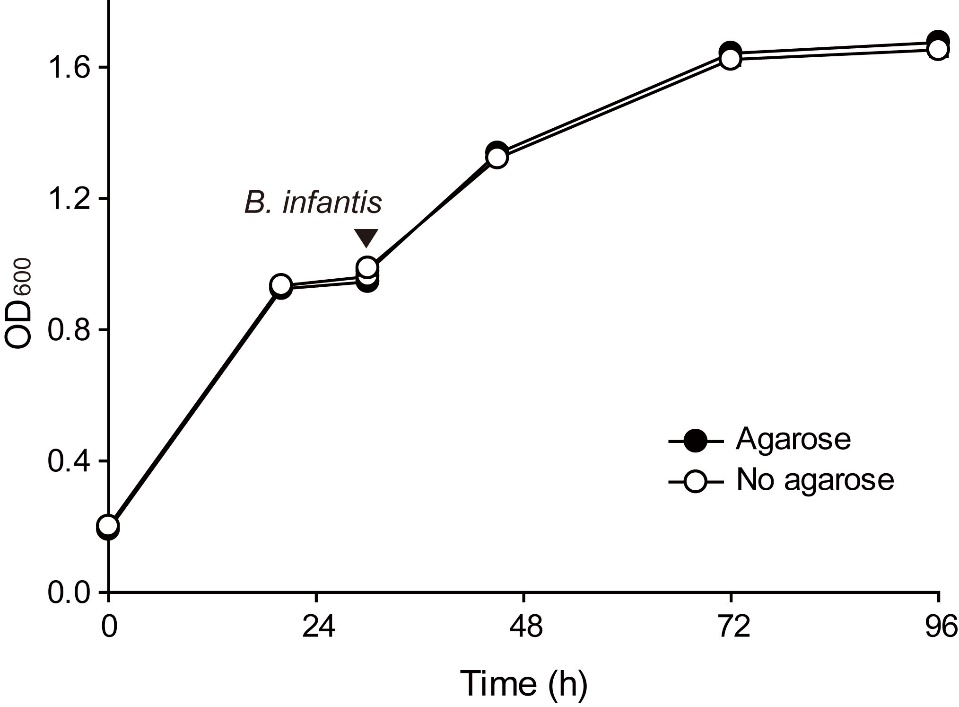


**Figure S9.** Growth profiles obtained from the sequential co-culture of *B. plebeius* and *B. infantis* grown in the presence of agarose (Agarose) or in the absence of agarose (No agarose). Error bars represent means ± S.D.
